# Supplementary material for: Adaptive Weight Selection for Time‐To‐Event Data Under Non‐Proportional Hazards
Source: Stat Med. 2025 Mar 17;44(6):e70045. doi: 10.1002/sim.70045 (PMC11912538; doi:10.1002/sim.70045)
Supplement: Supplementary file 1 — Supporting Information. Supporting Information. [file SIM-44-0-s001.pdf]

Adaptive multi-directional log rank test - Supplementary Material

Contents

A Technical appendix **2**

B Additional details to the real data example in Section ?? of the main manuscript **4**
B.1 Recruitment and censoring mechanism 4
B.2 Royston-Parmar spline fits for interim data 4
B.3 Conditional power calculations 8
B.4 Dependence from simulated recruitment data 10
B.5 Application of modestly weighted log-rank tests 11

C Additional simulation results **13**
C.1 Empirical type I error rates 13
C.2 Power comparisons 16
C.2.1 Additional deviation types 16
C.2.2 Model choice based on AIC 23
C.2.3 Modelwise choice of test statistics in the second stage 25

A Technical appendix

In this section of the Supplementary Material, we state some technical results that justify the validity of our adaptive weight selection procedure. We adopt the notation from the main manuscript.

Lemma 1. *Let $(\mathbf{X}^{(n)})_{n\geq 0}$ be a sequence of $\mathbb{R}^d$-valued random vectors s.t. $X_c^{(n)} \xrightarrow{\mathbb{P}} X_c$ for each $c \in \{1,\dots,d\}$ as $n \to \infty$. Then, it also holds*

$$\mathbf{X}^{(n)} \xrightarrow{\mathbb{P}} \mathbf{X} =: (X_1, \dots X_d)$$

as $n \to \infty$ in $\mathbb{R}^d$.

Proof. As all norms are equivalent on $\mathbf{R}^d$, it is enough to show it for the 1-norm, i.e.

$$\mathbb{P}\left[\sum_{c=1}^{d} |X_c^{(n)} - X_c| > \varepsilon\right] \to 0$$

for any $\varepsilon > 0$. Because $\sum_{c=1}^{d} |X_c^{(n)} - X_c| > \varepsilon$ implies that there is at least one c s.t. $|X_c^{(n)} - X_c| > \varepsilon/d$, we get

$$\begin{aligned}
&\mathbb{P}\left[\sum_{c=1}^{d} |X_c^{(n)} - X_c| > \varepsilon\right] \\
\leq &\mathbb{P}\left[\cup_{c=1}^{d} |X_c^{(n)} - X_c| > \varepsilon/d\right] \\
\leq &\sum_{c=1}^{d} \mathbb{P}[|X_c^{(n)} - X_c| > \varepsilon/d]
\end{aligned}$$

As all of the summands in the last sum converge to 0, the sum becomes arbitrary small for increasing n. □

Extending the notation from the manuscript, we define the bivariate $\mathbb{R}$-valued stochastic process $(T_{\hat{Q}}(t,s))_{t,s\geq 0}$ by

$$T_{\hat{Q}}(t,s) := n^{-\frac{1}{2}} \sum_{i=1}^{n} \int_{[0,s]} \hat{Q}(t,u) \left(Z_i - \frac{Y^{Z=1}(t,u)}{Y(t,u)}\right) dN_i(t,u),$$

that sums up the information available at calendar time t about the time-to-event endpoint until trial time s. This process has to be adapted to the bivariate filtration $(\mathcal{F}(t,s))_{t,s\geq 0}$ with

$$\mathcal{F}(t,s) = \sigma\left(\cup_{i=1}^{n} \mathcal{F}_i(t,s)\right),$$

i.e. these σ-algebras are generated by patient-specific σ-algebras. These $\mathcal{F}_i(t,s)$ are in turn generated by the random variables

$$\mathbb{1}_{\{R_i\leq t\}}, R_i \cdot \mathbb{1}_{\{R_i\leq t\}}, \mathbb{1}_{C_i^\star \leq s\wedge(t-R_i)_+}, C_i^\star \cdot \mathbb{1}_{C_i^\star \leq s\wedge(t-R_i)_+},$$
$$\mathbb{1}_{T_i\leq s\wedge C_i(t)}, T_i^{\cdot} \mathbb{1}_{T_i\leq s\wedge C_i(t)}.$$

The following results are valid under the null hypothesis of equal distributions of the time-to-event variable T

Theorem 1. *If for all $t \geq 0$, the assumptions*

(A1) For any $\tau < t$

$$\sup_{0\leq s\leq \tau} |\hat{Q}(t,s) - Q(t,s)| \xrightarrow{\mathbb{P}} 0$$

(A2) In its second argument, $\hat{Q}(t,s)$ is bounded over $[0,t]$, is left-continuous and has right hand limits

(A3) For any $\tau_1 > 0$ and $\tau_2 < t$

$$\sup_{\tau_1\leq s\leq \tau_2} \left|\frac{Y^{Z=1}(t,s)}{Y(t,s)} - \frac{y^{Z=1}(t,s)}{y(t,s)}\right| \xrightarrow{\mathbb{P}}$$

where $y(t,s) := \mathbb{E}[Y(t,s)]$ and $y^{Z=1}(t,s) := \mathbb{E}[Y^{Z=1}(t,s)]$

are fulfilled, then the process $(T_{\hat{Q}}(t))_{t\geq 0}$ *is asymptotically equivalent to the process* $(\tilde{T}_Q(t))_{t\geq 0}$*, i.e.*

$$(T_{\hat{Q}}(t) - \tilde{T}_Q(t)) \xrightarrow{\mathbb{P}} 0 \qquad \forall t \geq 0$$

This process is defined by

$$\tilde{T}_Q(t,s) := n^{-\frac{1}{2}} \sum_{i=1}^{n} \int_{[0,s]} Q(t,s) \left(Z_i - \frac{Y^{Z=1}(t,s)}{Y(t,s)} \right) dM_i(t,s),$$

where $(M_i(t,s))_{t,s\geq 0}$ *is the counting process martingale based on the counting process* $(N_i(t,s))_{t,s\geq 0}$.

For a proof, we refer to Theorem 1 of the Supplementary Material of[3]. In particular, it should be noted that the random quantities $\hat{Q}$, Y and $Y^{Z=1}$ have been replaced by deterministic quantities. Applying Lemma 1, we obtain the following Corollary.

Corollary 1. *The multivariate processes* $(\mathbf{T}_{\hat{\mathcal{Q}}}(t))_{t\geq 0}$ *as in Section* **??** *and the process* $(\tilde{\mathbf{T}}_{\mathcal{Q}}(t))_{t\geq 0}$*, where* $\mathcal{Q}$ *denotes the set of deterministic limit functions of those functions in* $\hat{\mathcal{Q}}$ *and* $\tilde{\mathbf{T}}_{\mathcal{Q}}(t) := (\tilde{T}_Q)_{Q\in\mathcal{Q}}$*, are asymptotically equivalent.*

Lemma 2. *If all functions* $Q \in \mathcal{Q}$ *and* $y^{Z=1}(t,s)/y(t,s)$ *are independent of their first arguments, the multivariate processes* $(\tilde{\mathbf{T}}_{\mathcal{Q}}(t))_{t\geq 0}$ *is a martingale w.r.t. the filtration* $(\mathcal{F}(t))_{t\geq 0}$ *that comprises all available information in calendar time, i.e.* $\mathcal{F}(t) := \mathcal{F}(t,t)$.

The proof follows analogously to the proof of Lemma 2 in Danzer et al. (2023)[3].
Please note that the assumptions made in this Lemma are naturally fulfilled in our applications. For Fleming-Harrington weights, the limit functions are given by $F(t)^\rho \cdot S(t)^\rho$ and under the null hypothesis $y^{Z=1}(t,s)/y(t,s)$ reduces to the (constant) probability that an individual is assigned to the treatment group.

Theorem 2. *As* $n \to \infty$, $(\tilde{\mathbf{T}}_{\mathcal{Q}}(t))_{t\geq 0}$ *converges in distribution to a Gaussian mean-zero vector martingale on some interval* $[0, t_{mas}]$ *with the* $|\mathcal{Q}| \times |\mathcal{Q}|$*-matrix-valued covariance function* $\mathbf{\Sigma}_{\mathcal{Q}} \colon [0, t_{max}] \to \mathbb{R}^{|\mathcal{Q}|\times|\mathcal{Q}|}$ *given by*

$$(\mathbf{\Sigma}(\boldsymbol{t}))_{kl} := \int_{[0,t]} Q_k(s) Q_l(s) \cdot \mathbb{P}[s \leq C(t) \wedge T] \cdot \mathbb{P}[Z=1](1-\mathbb{P}[Z=1]) dA(s).$$

The proof follows analogously to the proof of Theorem 2 in Danzer et al. (2023)[3]. The covariance function can be consistently estimated as stated in Section **??**.

Corollary 2. *For a sequence of analysis dates* $0 =: t_0 < t_1 < \cdots < t_m$ *in calendar time, the test multivariate test statistics* $\mathbf{T}_{\hat{\mathcal{Q}}}$ *are asymptotically jointly normally distributed with asymptotically independent increments, i.e.*

$$(\mathbf{T}_{\hat{\mathcal{Q}}}(t_1), \ldots, \mathbf{T}_{\hat{\mathcal{Q}}}(t_m)) \xrightarrow{\mathcal{D}} \mathcal{N}(0, \mathbf{\Sigma}_{\mathcal{Q},acc})$$
$$(\mathbf{T}_{\hat{\mathcal{Q}}}(t_1) - \mathbf{T}_{\hat{\mathcal{Q}}}(t_0), \ldots, \mathbf{T}_{\hat{\mathcal{Q}}}(t_m) - \mathbf{T}_{\hat{\mathcal{Q}}}(t_{m-1})) \xrightarrow{\mathcal{D}} \mathcal{N}(0, \mathbf{\Sigma}_{\mathcal{Q},inc})$$

where both $\mathbf{\Sigma}_{\mathcal{Q},acc}$ *and* $\mathbf{\Sigma}_{\mathcal{Q},inc}$ *are* $m|\mathcal{Q}| \times m|\mathcal{Q}|$ *matrices consisting of* m^2 *blocks of size* $|\mathcal{Q}| \times |\mathcal{Q}|$*. The block in row* r_1 *and column* r_2 *of* $\mathbf{\Sigma}_{\mathcal{Q},acc}$ *is given by* $\mathbf{\Sigma}_{\mathcal{Q}}(t_{r_1} \wedge t_{r_2})$ *and* $\mathbf{\Sigma}_{\mathcal{Q},inc}$ *is a block diagonal matrix with* $\mathbf{\Sigma}_{\mathcal{Q},inc} = diag(\mathbf{\Sigma}_{\mathcal{Q}}(t_1) - \mathbf{\Sigma}_{\mathcal{Q}}(t_0), \ldots, \mathbf{\Sigma}_{\mathcal{Q}}(t_m) - \mathbf{\Sigma}_{\mathcal{Q}}(t_{m-1}))$
Accordingly,

$$(\Psi_1(\mathbf{T}_{\hat{\mathcal{Q}}}(t_1) - \mathbf{T}_{\hat{\mathcal{Q}}}(t_0)), \ldots, \Psi_m(\mathbf{T}_{\hat{\mathcal{Q}}}(t_m) - \mathbf{T}_{\hat{\mathcal{Q}}}(t_{m-1})))$$

forms a set of asymptotically independent random variables for any set of Borel-measurable functions $\{\Psi_1, \ldots, \Psi_m\}$ *with* $\Psi_j \colon \mathbb{R}^{|\mathcal{Q}|} \to \mathbb{R}^{k_j}$ *for any* $k_j \in \mathbb{N}$ *for all* $j \in \{1, \ldots, m\}$.

As in Corollary 3 of Danzer et al. (2023), this is a consequence of the previous results.
The last statement allows us to apply the results and techniques of Brendel et al. (2014) and Ditzhaus & Friedrich (2019)[2;4] also to the increments of the multivariate test statistics.

B Additional details to the real data example in Section ?? of the main manuscript

In Section ?? of the main manuscript, we reanalysed reconstructed data from the FAKTION trial[6] (NCT number NCT01992952). Further details, that are also mentioned in the main manuscript, will be given here.

B.1 Recruitment and censoring mechanism

Here, we give some additional details on how and on what basis we reconstructed/simulated recruitment dates. Recruitment dates are required to apply administrative censoring at the interim analysis date to obtain hypothetical interim data.
According to the reconstructed data, the last observation has been censored at $t_2 := 35.98$ months, which is approximately 36 months. This corresponds to the total recruitment duration specified in the published manuscript[6]. Additionally, the hypothetical censoring distribution at the final analysis that emerges from a uniform recruitment over the whole trial duration is very similar to the estimated distribution from reconstructed data (see Figure S1). Hence, we assume that $C^\star = \infty$ with probability 1 and $R \sim \text{Unif}[0, t_2]$ according to the notation introduced in Section ??.

Therefore, we set $R_i = t_2 - X_i(t_2)$ if $\delta_i(t_2) = 0$ and simulate $R_i \sim [0, t_2 - X_i(t_2)]$ if $\delta_i(t_2) = 1$. This

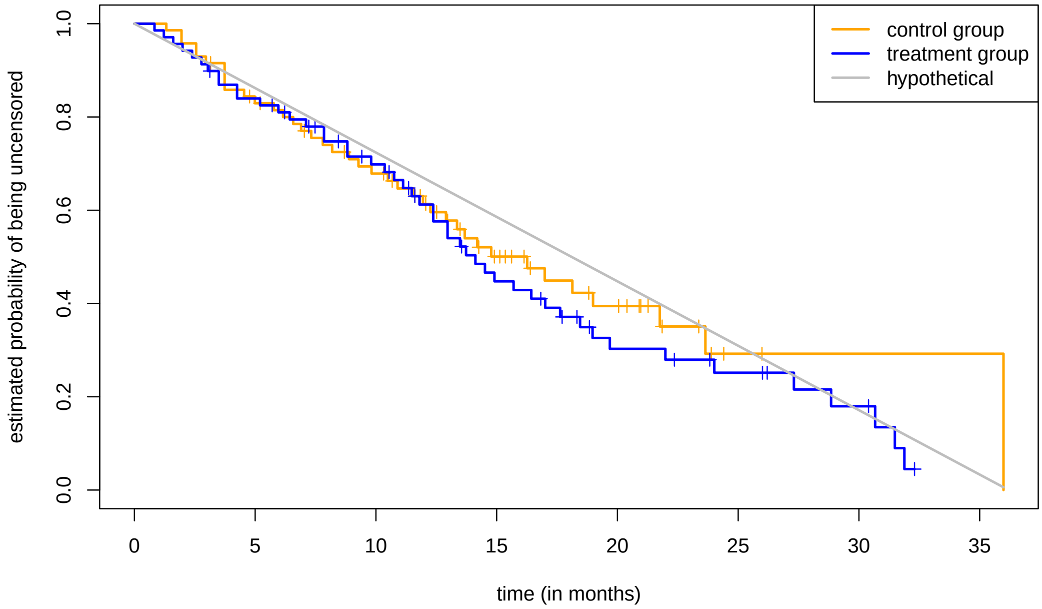

Supplementary Figure S1: Kaplan-Meier estimates of the survival function of the group-wise censoring distribution from reconstructed data. Grey line indicates hypothetical function if recruitment occurs uniformly over the whole trial duration and no additional loss to follow-up occurs.

simulation is in accordance with our preceding comments as $R|R \le r \sim \text{Unif}[0, r]$ for any uniformly distributed random variable R on $[0, r_{\max}]$ with $r \le r_{\max}$.
Obviously, the final results will depend on the simulated recruitment dates for uncensored observations. This dependence will be investigated further in Section B.4.

B.2 Royston-Parmar spline fits for interim data

After administrative censoring at calendar time $t_1 = 24$ was applied to the reconstructed and simulated data, the Kaplan-Meier estimates in Figure S2 can be obtained. Royston-Parmar splines were fitted to this data for each group separately using the function `flexsurvspline` from the R package `flexsurv`[5]. The Nelder-Mead optimization method has been chosen. Models with the number of interior knots

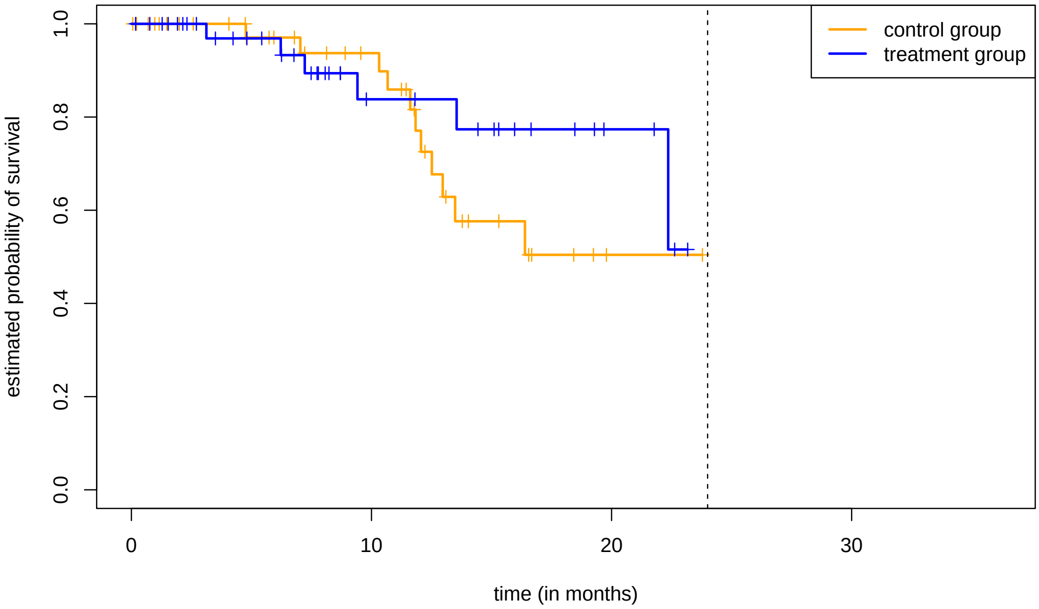

Supplementary Figure S2: Group-wise Kaplan-Meier estimates from interim data.

$p \in \{0, 1, 2\}$ and on all three available scales (hazard, odds, normal) were fitted. For each fit, the combined AIC was computed. The values can be found in Table S1. The lowest AIC is achieved for $p = 0$ on the normal scale. This results in a log-normal distribution[9]. It would also be possible to choose a different p and scale for the two groups. However, we restricted ourselves to the application of the same modeling parameters for both groups. On the next page, in Figure S3, the fitted Royston-Parmar spline models for all considered configurations are shown. On the page following afterwards (Figure S4), the Kaplan-Meier estimate based on the complete data is also shown in order to display the extrapolation performance.

		scale		
		hazard	odds	normal
p	0	160.31	158.65	157.97
	1	162.03	161.82	162.03
	2	160.70	161.04	161.51

Supplementary Table S1: AIC values for various Royston-Parmar spline models when fitted to the interim data

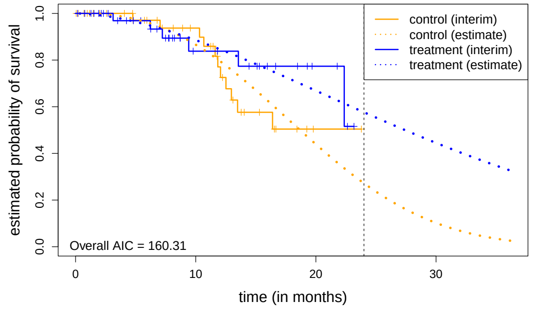

(a) $p = 0$, hazard scale

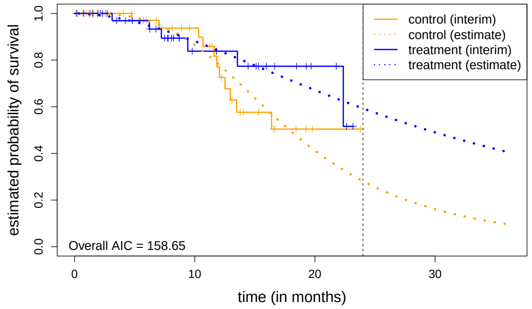

(b) $p = 0$, odds scale

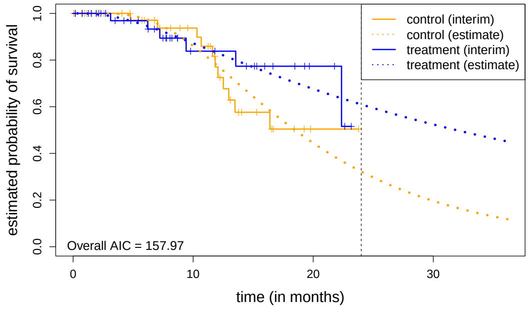

(c) $p = 0$, normal scale

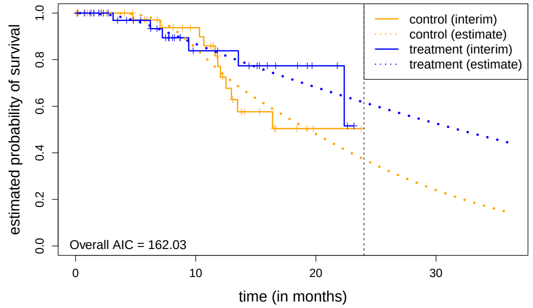

(d) $p = 1$, hazard scale

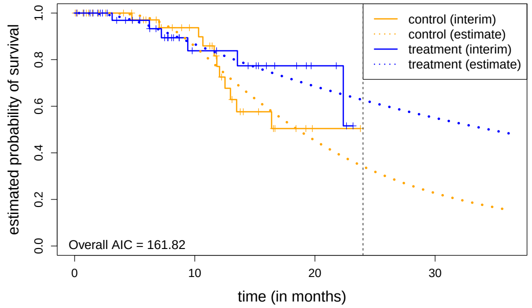

(e) $p = 1$, odds scale

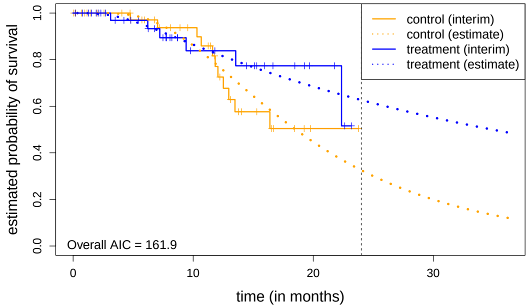

(f) $p = 1$, normal scale

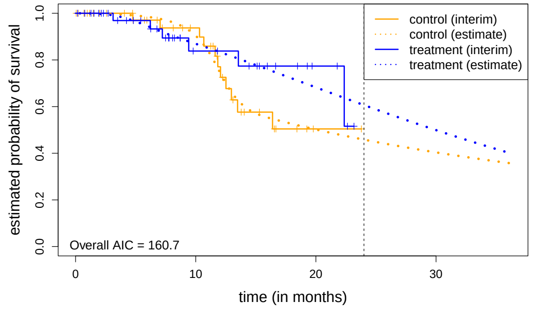

(g) $p = 2$, hazard scale

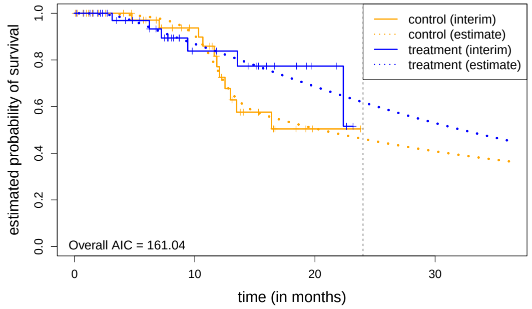

(h) $p = 2$, odds scale

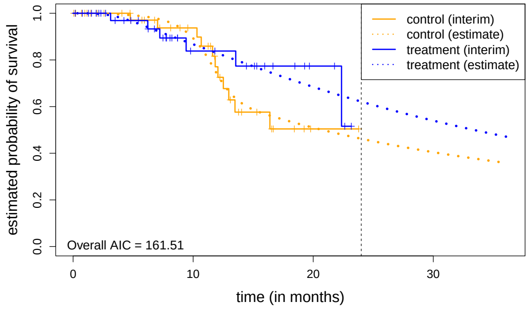

(i) $p = 2$, normal scale

Supplementary Figure S3: Fits of Royston-Parmar spline models to interim data.

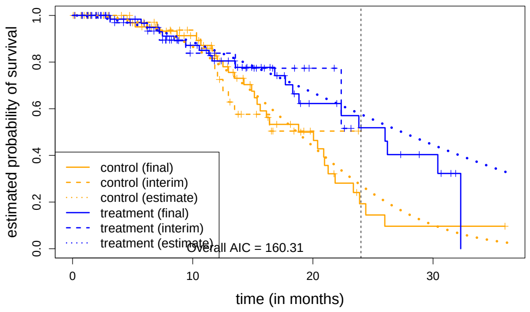

(a) $p = 0$, hazard scale

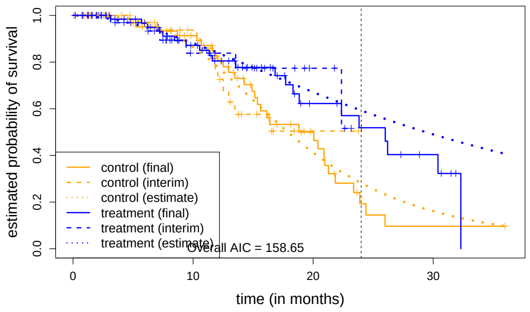

(b) $p = 0$, odds scale

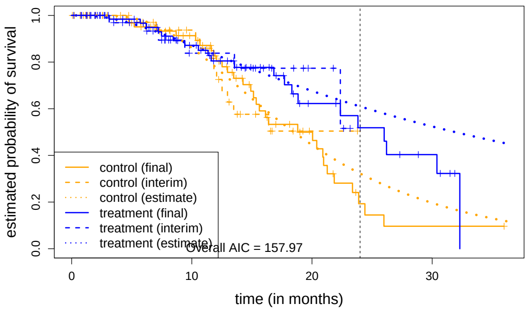

(c) $p = 0$, normal scale

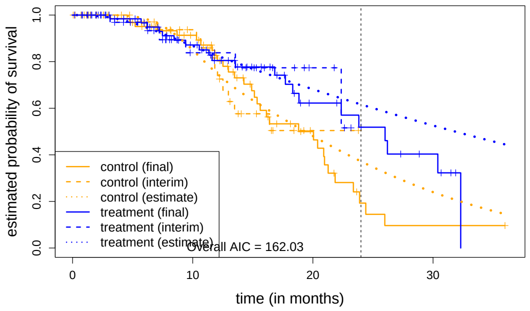

(d) $p = 1$, hazard scale

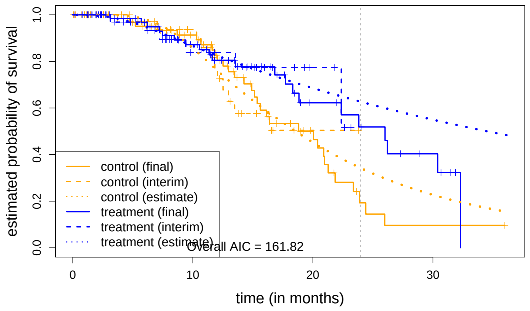

(e) $p = 1$, odds scale

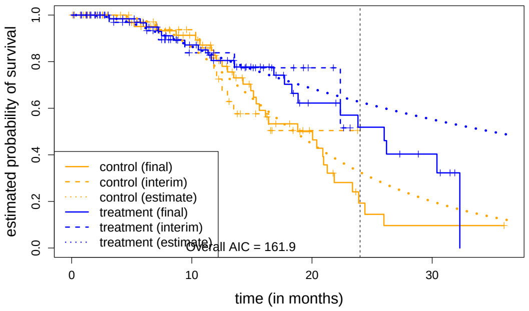

(f) $p = 1$, normal scale

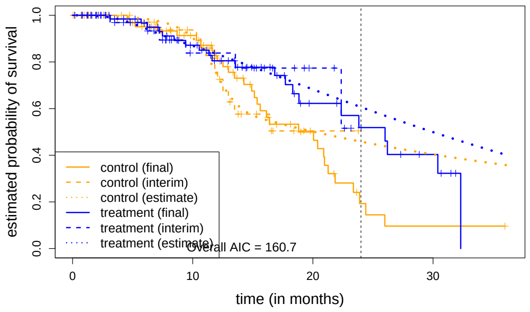

(g) $p = 2$, hazard scale

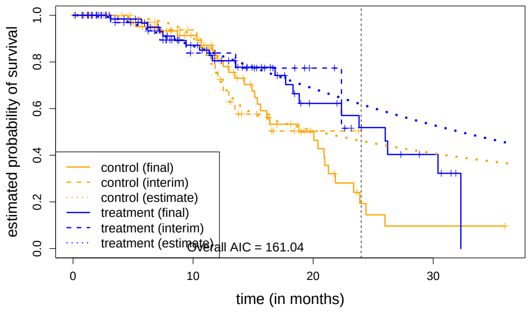

(h) $p = 2$, odds scale

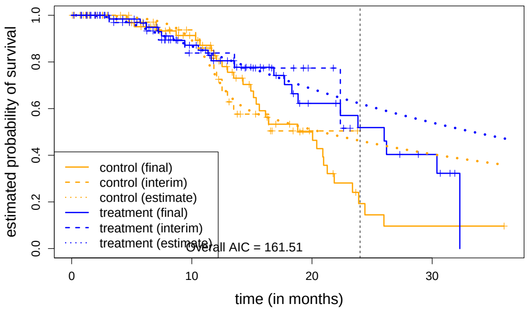

(i) $p = 2$, normal scale

Supplementary Figure S4: Fits of Royston-Parmar spline models to interim data with additional display of the final Kaplan-Meier estimates.

B.3 Conditional power calculations

Here, we present the results of the conditional power calculations. We consider

- 8 different mdir combination tests for the first stage (as presented in Table **??** in the main manuscript); the sets of weights used in these tests will be indexed as follows

$$\mathcal{Q}_{\mathbf{mdir},1} = \{(0,0),(1,0),(0,1),(1,1)\}$$
$$\mathcal{Q}_{\mathbf{mdir},2} = \{(0,0),(1,0),(0,1)\}$$
$$\mathcal{Q}_{\mathbf{mdir},3} = \{(0,0),(1,0),(1,1)\}$$
$$\mathcal{Q}_{\mathbf{mdir},4} = \{(0,0),(0,1),(1,1)\}$$
$$\mathcal{Q}_{\mathbf{mdir},5} = \{(0,0),(1,0)\}$$
$$\mathcal{Q}_{\mathbf{mdir},6} = \{(0,0),(0,1)\}$$
$$\mathcal{Q}_{\mathbf{mdir},7} = \{(0,0),(1,1)\}$$
$$\mathcal{Q}_{\mathbf{mdir},8} = \{(0,0)\}$$

- 9 different parameter constellations to fit Royston-Parmar splines to the interim data (number of knots $p \in \{0,1,2\}$, hazard, odds or normal scale)
- 4 different single-weighted tests for the second stage

For each combination of the mdir combination tests in the first stage and parameter constellation of the Royston-Parmar spline mode, the weighted test achieving the highest conditional power is marked in bold. Obviously, the choice does not depend on the first stage test statistic, as it is a monotone function of the standardized drift that is computed for each of the weighted tests in the second stage.
It is remarkable that for most of the choices of parameters for the Royston-Parmar splines (6 out of 9), the $(1,1)$-weighted test is favoured. This test actually has the best performance (see main manuscript). However, the test with the worst performance (weight $(1,0)$) is also chosen once. In the remainig two cases, the $(0,1)$-weighted test is favoured.

		hazard scale				odds scale				normal scale			
2nd stage weight		$(0,0)$	$(1,0)$	$(0,1)$	$(1,1)$	$(0,0)$	$(1,0)$	$(0,1)$	$(1,1)$	$(0,0)$	$(1,0)$	$(0,1)$	$(1,1)$
$p=0$	$\mathcal{Q}_{\mathrm{mdir},1}$	0.8089	0.6396	**0.8868**	0.87	0.7537	0.6233	0.8105	**0.8295**	0.7052	0.5649	0.7955	**0.8033**
	$\mathcal{Q}_{\mathrm{mdir},2}$	0.7653	0.5821	**0.8553**	0.8356	0.704	0.5651	0.7671	**0.7888**	0.6514	0.5052	0.7503	**0.7591**
	$\mathcal{Q}_{\mathrm{mdir},3}$	0.8307	0.6702	**0.9019**	0.8868	0.7791	0.6543	0.8321	**0.8497**	0.7331	0.5972	0.8182	**0.8255**
	$\mathcal{Q}_{\mathrm{mdir},4}$	0.8191	0.6538	**0.8939**	0.8779	0.7655	0.6376	0.8206	**0.839**	0.7182	0.5798	0.8061	**0.8137**
	$\mathcal{Q}_{\mathrm{mdir},5}$	0.7851	0.6076	**0.8698**	0.8513	0.7263	0.5908	0.7867	**0.8073**	0.6754	0.5314	0.7707	**0.7791**
	$\mathcal{Q}_{\mathrm{mdir},6}$	0.7411	0.5519	**0.8371**	0.8158	0.6769	0.5347	0.743	**0.7659**	0.6226	0.4746	0.7253	**0.7345**
	$\mathcal{Q}_{\mathrm{mdir},7}$	0.8384	0.6814	**0.9072**	0.8927	0.7882	0.6658	0.8398	**0.8569**	0.7433	0.6093	0.8264	**0.8334**
	$\mathcal{Q}_{\mathrm{mdir},8}$	0.7605	0.5759	**0.8517**	0.8316	0.6985	0.5588	0.7622	**0.7842**	0.6455	0.4989	0.7453	**0.7541**
$p=1$	$\mathcal{Q}_{\mathrm{mdir},1}$	0.598	0.4835	0.6715	**0.6932**	0.679	0.5493	0.7718	**0.7878**	0.74	0.5875	**0.8501**	0.85
	$\mathcal{Q}_{\mathrm{mdir},2}$	0.539	0.4241	0.6156	**0.6386**	0.6235	0.4895	0.7239	**0.7417**	0.6891	0.5282	**0.8123**	0.8123
	$\mathcal{Q}_{\mathrm{mdir},3}$	0.6297	0.5166	0.7009	**0.7217**	0.7081	0.5819	0.7961	**0.811**	0.7662	0.6194	**0.8686**	0.8685
	$\mathcal{Q}_{\mathrm{mdir},4}$	0.6126	0.4987	0.6852	**0.7065**	0.6925	0.5643	0.7831	**0.7987**	0.7522	0.6022	**0.8588**	0.8587
	$\mathcal{Q}_{\mathrm{mdir},5}$	0.565	0.45	0.6405	**0.663**	0.6482	0.5157	0.7455	**0.7625**	0.7119	0.5543	**0.8295**	0.8295
	$\mathcal{Q}_{\mathrm{mdir},6}$	0.5084	0.3942	0.5859	**0.6095**	0.594	0.4589	0.6977	**0.7163**	0.6615	0.4976	**0.791**	0.7909
	$\mathcal{Q}_{\mathrm{mdir},7}$	0.6415	0.5291	0.7117	**0.7321**	0.7188	0.5941	0.8048	**0.8194**	0.7757	0.6313	**0.8751**	0.8751
	$\mathcal{Q}_{\mathrm{mdir},8}$	0.5327	0.4179	0.6095	**0.6327**	0.6175	0.4832	0.7186	**0.7366**	0.6835	0.5219	**0.8081**	0.808
$p=2$	$\mathcal{Q}_{\mathrm{mdir},1}$	0.1992	**0.222**	0.1167	0.191	0.2345	0.2411	0.1687	**0.2442**	0.2532	0.2496	0.2033	**0.2743**
	$\mathcal{Q}_{\mathrm{mdir},2}$	0.1599	**0.1799**	0.0898	0.1528	0.1909	0.1968	0.1336	**0.1996**	0.2076	0.2044	0.1636	**0.2266**
	$\mathcal{Q}_{\mathrm{mdir},3}$	0.2231	**0.2474**	0.1338	0.2143	0.2607	0.2677	0.1904	**0.271**	0.2805	0.2767	0.2276	**0.3026**
	$\mathcal{Q}_{\mathrm{mdir},4}$	0.21	**0.2334**	0.1243	0.2015	0.2463	0.2531	0.1785	**0.2563**	0.2655	0.2618	0.2143	**0.2872**
	$\mathcal{Q}_{\mathrm{mdir},5}$	0.1764	**0.1977**	0.101	0.1688	0.2094	0.2156	0.1483	**0.2185**	0.227	0.2236	0.1803	**0.2469**
	$\mathcal{Q}_{\mathrm{mdir},6}$	0.1419	**0.1604**	0.078	0.1354	0.1707	0.1762	0.1177	**0.1788**	0.1864	0.1833	0.1453	**0.2042**
	$\mathcal{Q}_{\mathrm{mdir},7}$	0.2326	**0.2574**	0.1407	0.2236	0.2709	0.2781	0.199	**0.2814**	0.2911	0.2872	0.2371	**0.3136**
	$\mathcal{Q}_{\mathrm{mdir},8}$	0.1561	**0.1758**	0.0873	0.1491	0.1867	0.1925	0.1302	**0.1952**	0.2032	0.2	0.1597	**0.2219**

Supplementary Table S2: Conditional power for different choices of $\mathcal{Q}_{\mathrm{mdir}}$ single weighted log-rank test for the second stage test for different parameter configurations of the Royston-Parmar spline model

B.4 Dependence from simulated recruitment data

The results in Section ?? and in the previous subsections of course depend on the simulated recruitment data for patients with uncensored event time data.
Here, we want to assess the dependence from this simulation. Therefore, we repeat the procedure 10,000 times. Each time, interim data is analysed with the 8 different mdir combination tests mentioned above and 9 different Royston-Parmar spline models are fitted to the data. The second stage data is analysed with one of the for different single-weighted log-rank tests listed above. In Supplementary Table ??, the empirical power of the respective combinations of first- and second-stage tests is shown.

first stage test	second stage test			
	$(0,0)$	$(0,1)$	$(1,0)$	$(1,1)$
$\mathcal{Q}_{\text{mdir},1}$	0.5492	0.3827	0.3596	0.8997
$\mathcal{Q}_{\text{mdir},2}$	0.5297	0.3782	0.3478	0.8859
$\mathcal{Q}_{\text{mdir},3}$	0.5994	0.4228	0.4032	0.9147
$\mathcal{Q}_{\text{mdir},4}$	0.5380	0.3594	0.3355	0.8942
$\mathcal{Q}_{\text{mdir},5}$	0.5768	0.4169	0.3924	0.9044
$\mathcal{Q}_{\text{mdir},6}$	0.0769	0.0042	0.1570	0.6228
$\mathcal{Q}_{\text{mdir},7}$	0.5915	0.4040	0.3800	0.9108
$\mathcal{Q}_{\text{mdir},8}$	0.0890	0.0081	0.1770	0.7065

Supplementary Table S3: Empirical power of the simulation study for all combinations of first stage combination testing procedures and single-weighted log-rank testing procedures in the second stage.

Please note that the combination of $\mathcal{Q}_{\text{mdir},8}$ in the first stage and the $(0,0)$-weighted log-rank test in the second stage basically constitutes a two-stage standard log-rank test as in [10]. In about 9% of all simulation runs, this leads to a rejection although the originally applied simple one-stage standard log-rank leads to a rejection.
Power can strongly be increased if a different test is chosen in the first stage and the $(1,1)$-weighted log-rank test is chosen for the second stage. However, this is a retrospective assessment and one cannot guarantee a better choice for the first-stage test. Nevertheless, if a deviation from proportional hazards is anticipated, it is reasonable to consider a different test for the first stage.
For the second stage, we consider a choice based on an extrapolation with Royston-Parmar splines, a model selection based on AIC values and a consecutive conditional power calculation. In Supplementary Table S4, it is shown how often the 9 Royston-Parmar spline models used in this example so far are chosen based on the AIC values. Based on these choices, the second stage tests considered here, are

		scale		
		hazard	odds	normal
p	0	0.1869	0.1741	0.4637
	1	0.0258	0.0032	0.0541
	2	0.0824	0.0011	0.0087

Supplementary Table S4: Relative frequency with which the various Royston-Parmar spline models are selected based on the AIC

chosen with the following frequencies if $\mathcal{Q}_{\text{mdir},8}$ is applied in the first stage: $(0,0)$: 0.1755; $(1,0)$: 0.4293; $(0,1)$: 0.1258; $(1,1)$: 0.2694. It seems like the chosen model prefers the $(1,0)$-weighted test although the performance for the weight $(1,1)$ is much better according to Supplementary Table S3.
Finally, we can evaluate the performance of this procedure in terms of the power. This needs to be done separately for each first stage test statistic. The results can be found in Supplementary Table S5

first stage test	$\mathcal{Q}_{\text{mdir},1}$	$\mathcal{Q}_{\text{mdir},2}$	$\mathcal{Q}_{\text{mdir},3}$	$\mathcal{Q}_{\text{mdir},4}$	$\mathcal{Q}_{\text{mdir},5}$	$\mathcal{Q}_{\text{mdir},6}$	$\mathcal{Q}_{\text{mdir},7}$	$\mathcal{Q}_{\text{mdir},8}$
empirical power	0.4802	0.4699	0.5103	0.4613	0.5027	0.2836	0.4915	0.3057

Supplementary Table S5: Relative frequency with which the various Royston-Parmar spline models are selected based on the AIC

One can see that the power can be increased by about 6 percentage points even if the standard log-rank test is chosen in the first stage. If the first stage test statistic is chosen appropriately, it can even

raise to about 50%.
In Supplementary Figure S5, we show the empirical distribution of the maximal conditional power among the four tests for the second stage, computed based on the Royston-Parmar spline model with the lowest AIC. We restrict ourselves to the case that the weights in $\mathcal{Q}_{\text{mdir},1}$ have been chosen for the first stage. Correpsponding histograms for other choices for the first stage are very similar. We encounter a well-known problem in conditional power calculation in adaptive designs. This is characterised by the fact that the distribution of this variable tends towards extremes, i.e. very large and very small values[1]. It could therefore make sense to consider alternative concepts here.

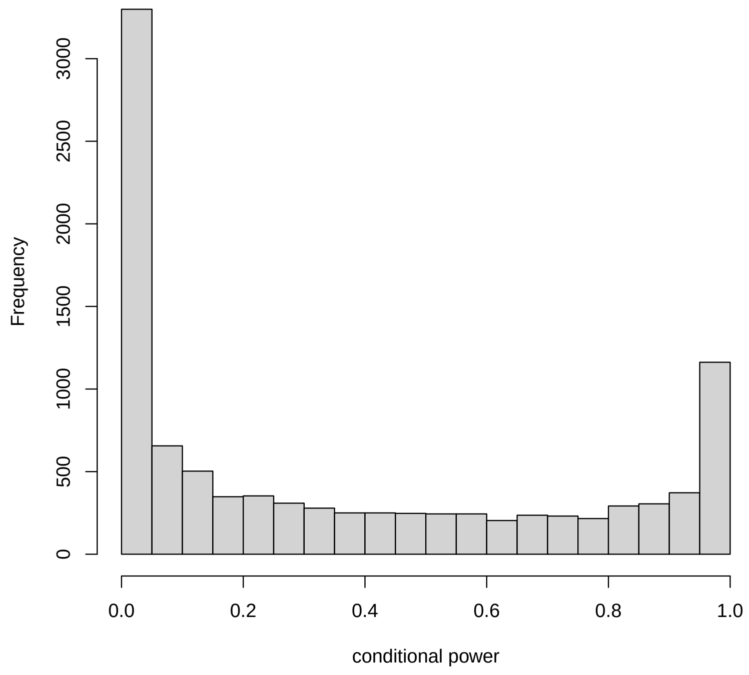

Supplementary Figure S5: Empirical distribution of the maximal conditional power that can be obtained for the four candidate tests for the second stage. The first stage test is an mdir combination test with weights in $\mathcal{Q}_{\text{mdir},1}$. The Royston-Parmar spline model is chosen based on the AIC.

B.5 Application of modestly weighted log-rank tests

As an additional analysis, we conduct a similar analysis to the one of Section B.4 with a different class of weights. The class of Fleming-Harrington weight has been criticized for its undesirable properties when applied in a one-sided testing procedure[8]. This applies equally when combining them in a combination testing procedure. In this context, Magirr and Burman suggested a different class of weights, which they termed "modest weights"[7].
The class of modest weights is parametrized by some threshold time $s^\star \geq 0$ and given by

$$\hat{Q}(t,s) = w_{\text{modest},s^\star}(\hat{S}(t,s-)) := \frac{1}{\max\left(\hat{S}(t,s-), \hat{S}(t,s^\star-)\right)}. \quad (1)$$

As the weights of Fleming and Harrington with $\rho > 0$ and $\gamma = 0$, this function is increasing in the second argument. Differently from these functions, it is bounded from below and it stays constant after the second argument exceeds the threshold $s^\star$. As previously shown, are an appropriate tool to detect late effects[7]. Notably, for $s^\star = 0$ the modestly weighted log-rank test is the same as the standard log-rank test as it is constantly equal to 1. The modest weights also fulfill the assumptions of Theorem 1 and can hence also be incorporated into our framework.
Here, we consider designs where a single modestly weighted log-rank test is applied in the first stage and

the second test stage is chosen among the modestly weighted tests with the thresholds $s^\star \in \{0, 3, 6, 9, 12, 15, 18, 21, 24\}$. The modest weights could also be used in a combination testing procedure as e.g. the *mdir* test. However, we do not consider this option here.

As previously, 10,000 simulation runs are made where the unreconstructable recruitment dates are simulated. Conditional power calculations for the 9 different modestly weighted tests are made from the same 9 different Royston-Parmar spline models as above. The best spline model is chosen based on the AIC and the second stage test is chosen as the modestly weighted test with the highest conditional power according to this spline model. The results in terms of the empirical power of the procedure in dependence from the (fixed) test in the first stage are displayed in Figure S6.

For comparison, the empirical rejection rate for the two-stage procedure which applies the standard

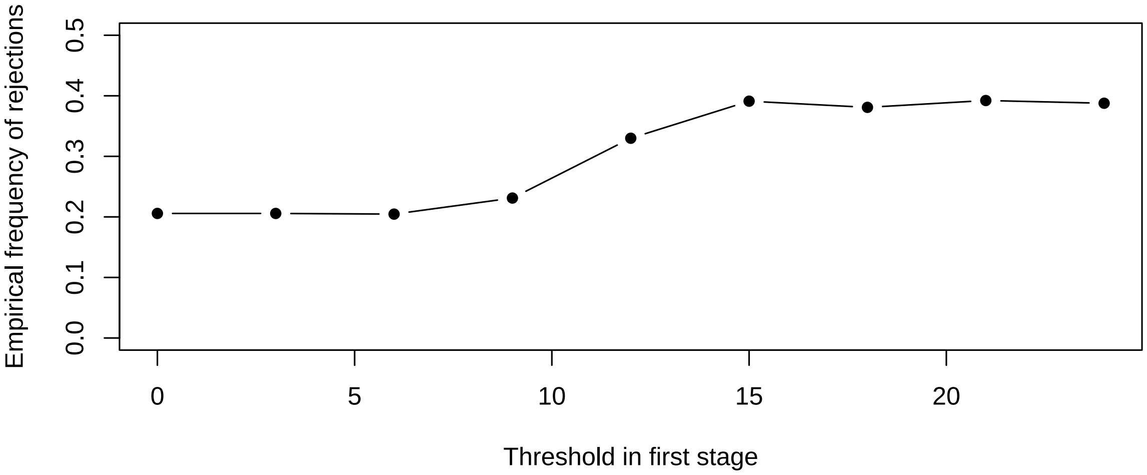

Supplementary Figure S6: Empirical power for an adpaitve design with a modestly weighted log-rank test in the first stage and a modestly weighted log-rank test in the second stage that is chosen based on conditional power consideration based on Royston-Parmar spline models. 9 different modestly weighted log-rank tests are considered. The threshold for the (fixed) test in the first stage is given by the x-axis.

log-rank test in both stages (i.e. $s^\star = 0$) is 9.31%. It is obvious that the selection procedure increases the rejection rate, even if the standard log-rank test is chosen for the first stage. The increase is even more articulate if a better-suited test is chosen in the first stage.

C Additional simulation results

In this section, we provide additional results to the simulation study in Section **??** of the main manuscript.

C.1 Empirical type I error rates

Here, we present the empirical type I error rates that emerge from fixed (in particular non-adaptive) combinations of testing procedures in the two stages. This shall demonstrate that our selection procedure (see Table **??**) induces no additional type I error rate inflation.
For the sake of presentability, we use the following numbering system for the various tests:

1: Standard log-rank test

2: Weighted log-rank test with Fleming-Harrington weight $w^{(0,1)} \circ \hat{F}$

3: Weighted log-rank test with Fleming-Harrington weight $w^{(0,2)} \circ \hat{F}$

4: Weighted log-rank test with Fleming-Harrington weight $w^{(0,3)} \circ \hat{F}$

5: Weighted log-rank test with Fleming-Harrington weight $w^{(1,1)} \circ \hat{F}$

6: Weighted log-rank test with Fleming-Harrington weight $w^{(1,0)} \circ \hat{F}$

7: Weighted log-rank test with Fleming-Harrington weight $w^{(2,0)} \circ \hat{F}$

8: Weighted log-rank test with Fleming-Harrington weight $w^{(3,0)} \circ \hat{F}$

9: *mdir* combination test based on the set of weights $\{w^{(0,0)} \circ \hat{F}, w^{(1,0)} \circ \hat{F}\}$

10: *mdir* combination test based on the set of weights $\{w^{(0,0)} \circ \hat{F}, w^{(1,1)} \circ \hat{F}\}$

11: *mdir* combination test based on the set of weights $\{w^{(0,0)} \circ \hat{F}, w^{(0,1)} \circ \hat{F}\}$

12: *mdir* combination test based on the set of weights $\{w^{(0,0)} \circ \hat{F}, w^{(1,0)} \circ \hat{F}, w^{(0,1)} \circ \hat{F}\}$

13: *mdir* combination test based on the set of weights $\{w^{(0,0)} \circ \hat{F}, w^{(1,0)} \circ \hat{F}, w^{(1,1)} \circ \hat{F}\}$

14: *mdir* combination test based on the set of weights $\{w^{(0,0)} \circ \hat{F}, w^{(1,1)} \circ \hat{F}, w^{(0,1)} \circ \hat{F}\}$

15: *mdir* combination test based on the set of weights $\{w^{(0,0)} \circ \hat{F}, w^{(1,0)} \circ \hat{F}, w^{(1,1)} \circ \hat{F}, w^{(0,1)} \circ \hat{F}\}$

	1	2	3	4	5	6	7	8	9	10	11	12	13	14	15
1	0.028	0.029	0.0302	0.0317	0.0274	0.027	0.0263	0.0259	0.0241	0.0255	0.0259	0.0254	0.0255	0.0262	0.0251
2	0.0301	0.0302	0.0316	0.0323	0.0308	0.0292	0.0289	0.0282	0.0279	0.028	0.0275	0.0274	0.0275	0.0273	0.0278
3	0.0313	0.0318	0.032	0.0337	0.0311	0.0306	0.0305	0.0305	0.0279	0.0289	0.0294	0.0278	0.0275	0.0287	0.028
4	0.0315	0.0319	0.0328	0.034	0.0317	0.0297	0.0305	0.0312	0.0286	0.0298	0.0293	0.0287	0.0294	0.0299	0.0284
5	0.0292	0.0292	0.0292	0.0301	0.0286	0.0273	0.0272	0.0278	0.0262	0.0267	0.0272	0.0275	0.0264	0.0268	0.0265
6	0.0269	0.0273	0.0302	0.0313	0.0269	0.0263	0.025	0.025	0.0256	0.0253	0.0259	0.0264	0.0252	0.0263	0.0258
7	0.0267	0.0281	0.0297	0.0315	0.0272	0.0254	0.0245	0.0245	0.0249	0.0255	0.0255	0.0255	0.0252	0.0253	0.0259
8	0.0269	0.0277	0.03	0.0317	0.0269	0.0253	0.0252	0.0244	0.0249	0.0257	0.0257	0.0259	0.0253	0.0261	0.0262
9	0.0258	0.0268	0.0274	0.0283	0.0255	0.025	0.0236	0.0237	0.0227	0.0236	0.0232	0.0234	0.0233	0.0236	0.0234
10	0.0267	0.0275	0.0278	0.0285	0.0261	0.0257	0.0253	0.0256	0.0231	0.0241	0.0248	0.0242	0.0241	0.0251	0.0246
11	0.0253	0.0258	0.0288	0.0304	0.0262	0.0251	0.0241	0.0235	0.0237	0.0239	0.0234	0.0234	0.0234	0.0229	0.0236
12	0.0257	0.0272	0.0282	0.0291	0.0265	0.0252	0.024	0.0243	0.0234	0.0238	0.0237	0.0232	0.0236	0.0246	0.0237
13	0.0263	0.0268	0.0276	0.0275	0.0267	0.0257	0.0245	0.0254	0.0232	0.0241	0.0242	0.0241	0.0238	0.025	0.024
14	0.0256	0.0268	0.0282	0.0299	0.0266	0.0262	0.0245	0.0244	0.0238	0.0248	0.0242	0.0237	0.0241	0.0245	0.0246
15	0.0258	0.027	0.0274	0.0287	0.0266	0.0244	0.0242	0.0249	0.0227	0.0235	0.0239	0.0231	0.0232	0.0252	0.0232

Supplementary Table S6: Empirical type I error rates for fixed combinations of stagewise tests with 50 patients in each group. Rows correspond to first-stage tests and columns correspond to second-stage tests.

	1	2	3	4	5	6	7	8	9	10	11	12	13	14	15
1	0.0283	0.0273	0.0278	0.0272	0.0285	0.0281	0.0283	0.0264	0.0259	0.0282	0.0279	0.0279	0.0266	0.0285	0.0275
2	0.0264	0.0274	0.0258	0.0258	0.0269	0.0261	0.0267	0.026	0.0248	0.0257	0.0258	0.0269	0.0265	0.0267	0.027
3	0.0272	0.0258	0.0271	0.028	0.0275	0.0271	0.0254	0.0256	0.0259	0.0274	0.0271	0.0288	0.0277	0.0277	0.029
4	0.0281	0.0264	0.0273	0.0283	0.0284	0.0275	0.0258	0.0261	0.0278	0.0287	0.0286	0.0285	0.029	0.0283	0.0288
5	0.026	0.0275	0.027	0.0273	0.0272	0.0266	0.0276	0.0265	0.0256	0.0262	0.026	0.0274	0.0265	0.0277	0.0267
6	0.0276	0.0266	0.0267	0.0263	0.0283	0.0294	0.0277	0.0277	0.025	0.0277	0.0282	0.0276	0.0262	0.0285	0.027
7	0.0278	0.0262	0.0266	0.0266	0.0283	0.0303	0.0294	0.0278	0.0264	0.0268	0.0294	0.0277	0.0266	0.0289	0.0278
8	0.0276	0.026	0.0259	0.0264	0.0279	0.03	0.0282	0.0287	0.0264	0.0276	0.0287	0.0285	0.0258	0.0279	0.0272
9	0.0261	0.0251	0.026	0.0253	0.0272	0.026	0.0258	0.0244	0.0243	0.0264	0.0265	0.027	0.0263	0.0269	0.0267
10	0.0268	0.0269	0.0261	0.0246	0.0283	0.0265	0.0263	0.0245	0.0245	0.0278	0.0266	0.0265	0.0262	0.0273	0.0258
11	0.0256	0.0263	0.0262	0.0257	0.027	0.0279	0.0272	0.0263	0.0239	0.0261	0.0275	0.0266	0.0248	0.0279	0.0252
12	0.027	0.0249	0.0264	0.0267	0.0276	0.0272	0.0269	0.0247	0.0241	0.0264	0.0272	0.0271	0.0256	0.0276	0.027
13	0.0264	0.0261	0.026	0.0252	0.0271	0.0263	0.0262	0.0256	0.0244	0.0272	0.0262	0.0272	0.0264	0.0269	0.0267
14	0.0273	0.0263	0.0266	0.0258	0.0291	0.0284	0.0275	0.0256	0.025	0.0281	0.0283	0.0278	0.0264	0.0283	0.0273
15	0.0271	0.0264	0.0258	0.0257	0.0276	0.0277	0.0258	0.0251	0.0237	0.0269	0.0275	0.0272	0.0256	0.0277	0.0261

Supplementary Table S7: Empirical type I error rates for fixed combinations of stagewise tests with 100 patients in each group. Rows correspond to first-stage tests and columns correspond to second-stage tests.

	1	2	3	4	5	6	7	8	9	10	11	12	13	14	15
1	0.0228	0.0211	0.0214	0.0218	0.0232	0.024	0.0236	0.0238	0.0224	0.0231	0.0233	0.0231	0.023	0.0231	0.0231
2	0.0257	0.0243	0.0234	0.0237	0.0249	0.0254	0.024	0.0261	0.025	0.0255	0.0254	0.0256	0.0246	0.0241	0.0251
3	0.0262	0.0252	0.0251	0.0249	0.026	0.0258	0.0262	0.0274	0.0255	0.0252	0.026	0.0256	0.0247	0.0255	0.0259
4	0.0261	0.026	0.0255	0.026	0.0268	0.0264	0.0264	0.0276	0.0271	0.0262	0.027	0.0272	0.026	0.0266	0.027
5	0.0237	0.0221	0.0222	0.0229	0.0242	0.0248	0.0246	0.0247	0.0225	0.0238	0.0229	0.0235	0.0224	0.0242	0.0239
6	0.0244	0.0216	0.0216	0.0218	0.0237	0.0239	0.0237	0.0237	0.0225	0.0245	0.0245	0.0233	0.0222	0.0237	0.0243
7	0.024	0.0234	0.0228	0.0227	0.0239	0.0236	0.024	0.0223	0.0235	0.0248	0.0241	0.0243	0.023	0.0242	0.0234
8	0.0242	0.0224	0.0225	0.0228	0.0233	0.0236	0.0226	0.0228	0.0232	0.024	0.0239	0.0238	0.0235	0.0239	0.0228
9	0.0237	0.0229	0.0221	0.0226	0.0236	0.0245	0.0244	0.0246	0.0227	0.0242	0.0238	0.024	0.0227	0.024	0.0241
10	0.0219	0.0217	0.021	0.0212	0.0233	0.0237	0.0237	0.0241	0.0225	0.0223	0.023	0.0227	0.0218	0.023	0.0227
11	0.0231	0.0218	0.0221	0.0222	0.0225	0.0248	0.0236	0.0241	0.0218	0.0238	0.0238	0.0237	0.0223	0.0239	0.0237
12	0.0234	0.0213	0.0213	0.0224	0.0237	0.0239	0.0247	0.0241	0.0217	0.0239	0.0239	0.0231	0.022	0.0239	0.0235
13	0.0237	0.0228	0.0216	0.0228	0.0245	0.0235	0.0253	0.025	0.0233	0.024	0.0238	0.0235	0.023	0.0245	0.0238
14	0.023	0.0217	0.021	0.0219	0.0244	0.0251	0.0245	0.0239	0.0228	0.0246	0.0237	0.0228	0.0226	0.0228	0.023
15	0.0239	0.0224	0.0214	0.0223	0.0233	0.0242	0.0255	0.0243	0.0223	0.0239	0.024	0.0235	0.0229	0.0243	0.0236

Supplementary Table S8: Empirical type I error rates for fixed combinations of stagewise tests with 200 patients in each group. Rows correspond to first-stage tests and columns correspond to second-stage tests.

	1	2	3	4	5	6	7	8	9	10	11	12	13	14	15
1	0.0263	0.0286	0.0288	0.029	0.0269	0.026	0.0254	0.0258	0.0277	0.027	0.0269	0.0278	0.0284	0.028	0.0282
2	0.0252	0.0279	0.0272	0.0275	0.0275	0.0257	0.025	0.0247	0.0271	0.0264	0.0243	0.025	0.0269	0.0256	0.0268
3	0.0263	0.0255	0.0259	0.0254	0.0265	0.0248	0.0247	0.0239	0.0261	0.0262	0.025	0.0252	0.0261	0.025	0.0262
4	0.0271	0.0266	0.0266	0.0265	0.0257	0.0235	0.0257	0.0251	0.0256	0.0276	0.0259	0.0259	0.0268	0.0258	0.0269
5	0.0255	0.0277	0.0283	0.0279	0.0273	0.0263	0.0263	0.0254	0.0263	0.0257	0.025	0.0256	0.0271	0.0256	0.0256
6	0.0279	0.0298	0.0286	0.0291	0.0288	0.0263	0.026	0.0256	0.0286	0.0288	0.0272	0.0284	0.0294	0.0284	0.0284
7	0.0276	0.0286	0.0279	0.0281	0.0285	0.0278	0.0269	0.0267	0.0282	0.0285	0.0278	0.028	0.0291	0.0284	0.0287
8	0.0275	0.0277	0.0271	0.0276	0.0284	0.027	0.027	0.0258	0.0282	0.0278	0.0268	0.0269	0.0277	0.0272	0.0276
9	0.0259	0.0285	0.0288	0.0286	0.0278	0.0261	0.0271	0.0261	0.0264	0.0266	0.0256	0.0269	0.0273	0.0262	0.0274
10	0.026	0.0274	0.0276	0.0276	0.0264	0.0258	0.0262	0.0256	0.0267	0.0265	0.0251	0.0269	0.0266	0.0259	0.0266
11	0.0274	0.0287	0.0285	0.0289	0.0286	0.0269	0.0264	0.0258	0.0281	0.0274	0.0267	0.0276	0.0284	0.0269	0.0266
12	0.0253	0.0276	0.0281	0.0272	0.0263	0.0258	0.0264	0.0269	0.0258	0.0267	0.0252	0.0261	0.0267	0.0254	0.0258
13	0.0253	0.0274	0.0275	0.0273	0.0265	0.025	0.0266	0.0264	0.0259	0.0263	0.0252	0.0256	0.0271	0.0261	0.0266
14	0.0265	0.028	0.0278	0.0275	0.0265	0.0258	0.0254	0.0262	0.0276	0.0266	0.0254	0.0265	0.0274	0.0255	0.0267
15	0.0255	0.0278	0.0272	0.027	0.0274	0.0257	0.0261	0.0259	0.0268	0.0265	0.025	0.0262	0.0265	0.0261	0.0258

Supplementary Table S9: Empirical type I error rates for fixed combinations of stagewise tests with 500 patients in each group. Rows correspond to first-stage tests and columns correspond to second-stage tests.

C.2 Power comparisons

C.2.1 Additional deviation types

In this subsection, we supplement the results from Section **??** of the main manuscript by those results for the settings $(\rho^\star, \gamma^\star) \in \{(0,1), (0,3), (1,0), (3,0)\}$. The corresponding survival curves are shown in Figure S7.

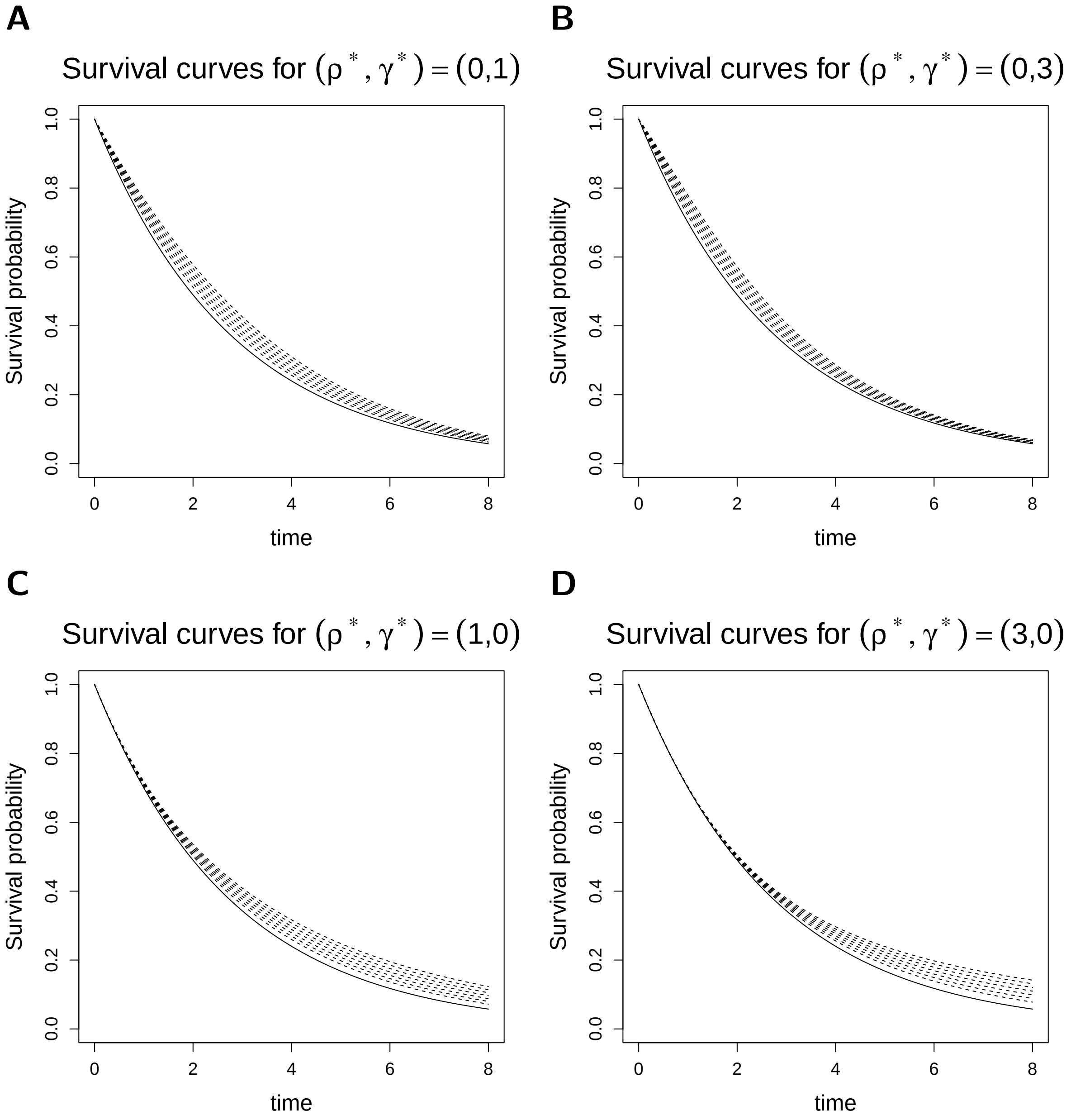

Supplementary Figure S7: Survival curves for three types of deviation of the distribution in the experimental group from the distribution in the control group. The survival curve in the control group is given by the solid line. Dashed lines are survival curves in the experimental group for the seven effect sizes $\{0.4 \cdot \theta_0, 0.6 \cdot \theta_0, 0.8 \cdot \theta_0, \theta_0, 1.2 \cdot \theta_0, 1.4 \cdot \theta_0, 1.6 \cdot \theta_0\}$. A) Survival curves in the slightly early effect case ($(\rho^\star, \gamma^\star) = (0,1)$) B) Survival curves in the very early effect case ($(\rho^\star, \gamma^\star) = (0,3)$) C) Survival curves in the slightly late effect case ($(\rho^\star, \gamma^\star) = (1,0)$) B) Survival curves in the very late effect case ($(\rho^\star, \gamma^\star) = (3,0)$)

For these scenarios, the power curves and the bar plots showing the choices made by the procedure **TS-AD** for the second stage test can be found in Figures S8-S12. For slightly early and late effects (i.e. $(\rho^\star, \gamma^\star) = (0, 1)$ or $(\rho^\star, \gamma^\star) = (1, 0)$, respectively) we can see that all procedures perform very similarly. For strong early and late effects (i.e. $(\rho^\star, \gamma^\star) = (0, 3)$ or $(\rho^\star, \gamma^\star) = (3, 0)$, respectively), there is a marked gap between the optimal two-stage test and the two-stage standard log-rank test. The one-stage combination testing procedures and the adaptive procedures fill this gap. Once again, we can see that the two restricted procedures with pre-chosen sets of weights perform better than the unrestricted ones. It should be noted that our selection procedure tends to favor the log-rank test with weights $w^{(0,3)} \circ \hat{F}$ in the case of early effects and the log-rank test with weights $w^{(1,0)} \circ \hat{F}$ in the case of late effects. This shows that there is certainly room for improvement in this selection process.

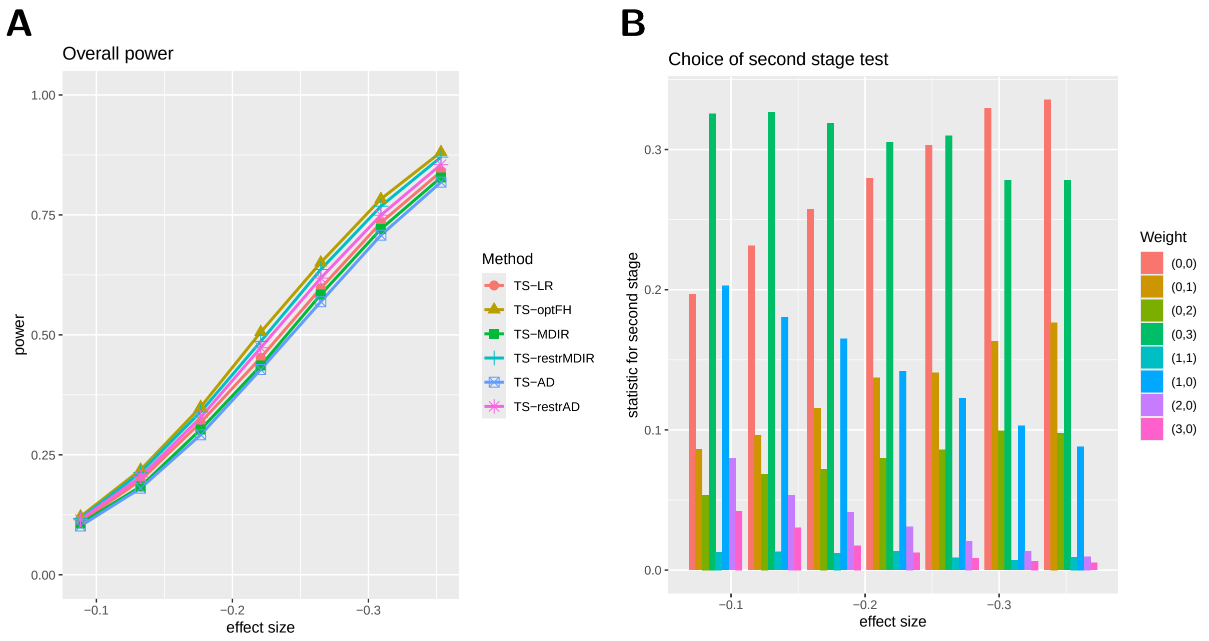

Supplementary Figure S8: A) Power curve for six testing procedures in case of slight early effect ($(\rho^\star, \gamma^\star) = (0, 1)$). Please note that the two procedures TS-AD and TS-optFH as well as the two procedures OS-MDIR and OS-restrMDIR coincide in this case. B) Relative frequencies of the choice of single weighted tests for the second stage for the testing procedure TS-AD.

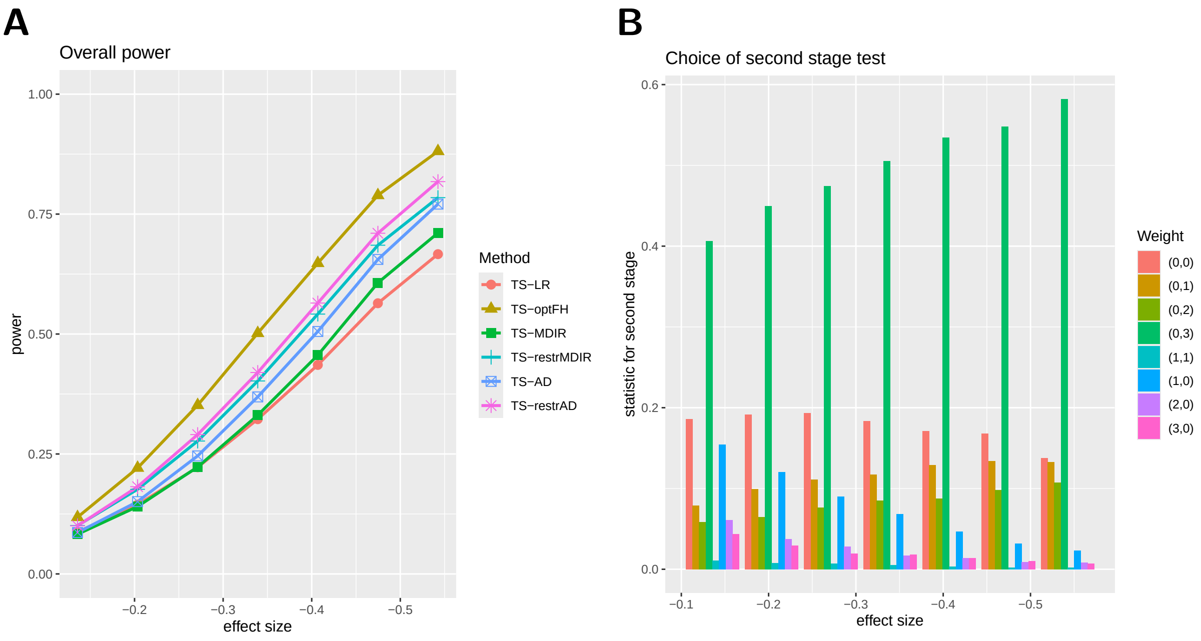

Supplementary Figure S9: A) Power curve for six testing procedures in pronounced early effect ($(\rho^\star, \gamma^\star) = (0, 3)$). Please note that the two procedures TS-AD and TS-optFH as well as the two procedures OS-MDIR and OS-restrMDIR coincide in this case. B) Relative frequencies of the choice of single weighted tests for the second stage for the testing procedure TS-AD.

Abbreviation	Description
**OS-MDIR**	One-stage testing procedure with the *mdir* combination test based on the weights $w^{(0,0)} \circ \hat{F}$, $w^{(1,0)} \circ \hat{F}$ and $w^{(0,1)} \circ \hat{F}$
**OS-restrMDIR**	One-stage testing procedure with an *mdir* combination test with a restricted set of weights in some cases ($w^{(0,0)} \circ \hat{F}$, $w^{(1,0)} \circ \hat{F}$ if $\rho^\star > \gamma^\star = 0$ and $w^{(0,0)} \circ \hat{F}$, $w^{(0,1)} \circ \hat{F}$ if $\gamma^\star > \rho^\star = 0$)
**OS-AD**	Adaptive design without early stopping with a *mdir* combination test as for OS-MDIR in the first stage and a selection of the test for the second stage among the weights in $\hat{\mathcal{Q}}_{\text{cand}}$ as defined above
**OS-LR**	One-stage standard log-rank test
**OS-optFH**	One-stage weighted log-rank test with the optimal weighting by $w^{(\rho^\star,\gamma^\star)} \circ \hat{F}$
**OS-restrAD**	Adaptive design without early stopping with a *mdir* combination test as for the design OS-restrMDIR in the first stage and a selection of the test for the second stage among a restricted subset of $\hat{\mathcal{Q}}_{\text{cand}}$ that only includes weights with $\rho \gtrless \gamma$ and the standard weight if $\rho^\star \gtrless \gamma^\star$ and weights with $\lvert \rho - \gamma \rvert \leq 1$ if $\rho^\star = \gamma^\star = 0$.

Supplementary Table S10: Overview of the different one-stage testing procedures.

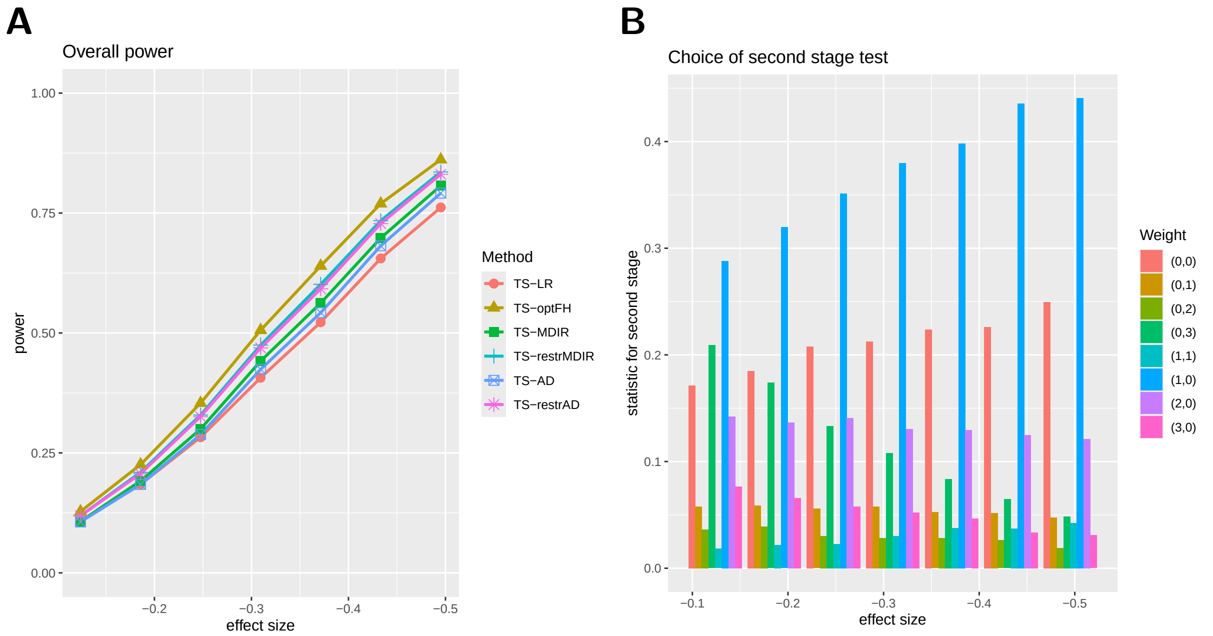

Supplementary Figure S10: A) Power curve for six testing procedures in case of slight late effect $((\rho^\star, \gamma^\star) = (1, 0))$. Please note that the two procedures TS-AD and TS-optFH as well as the two procedures OS-MDIR and OS-restrMDIR coincide in this case. B) Relative frequencies of the choice of single weighted tests for the second stage for the testing procedure TS-AD.

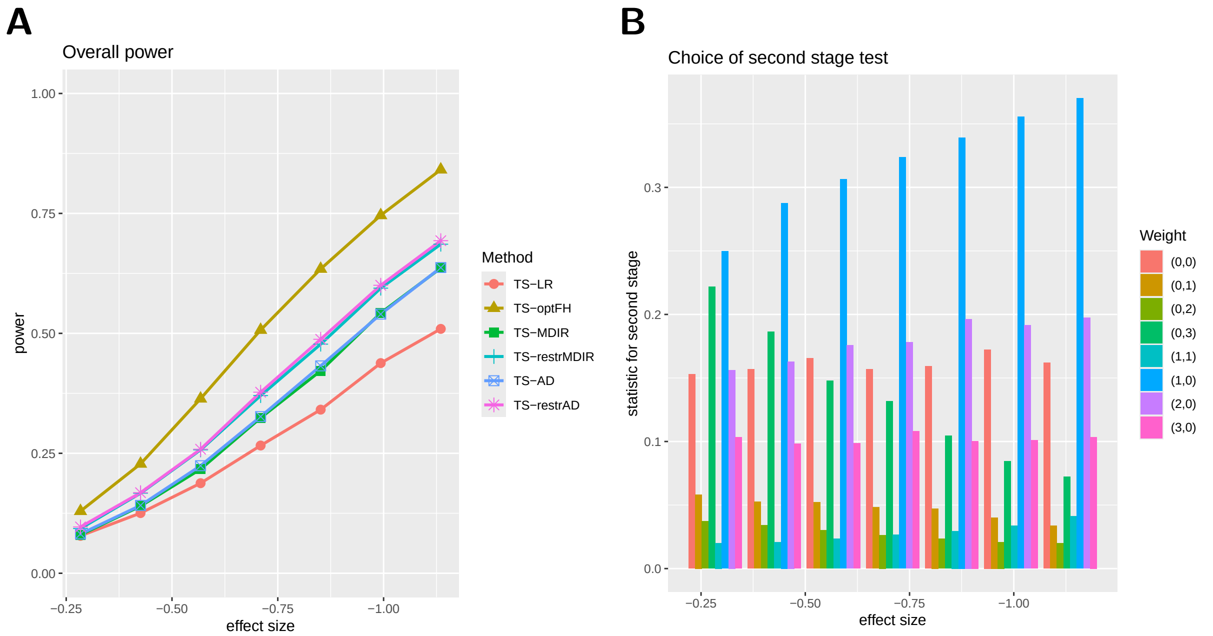

Supplementary Figure S11: A) Power curve for six testing procedures in case of pronounced late effect $((\rho^\star, \gamma^\star) = (3, 0))$. Please note that the two procedures TS-AD and TS-optFH as well as the two procedures OS-MDIR and OS-restrMDIR coincide in this case. B) Relative frequencies of the choice of single weighted tests for the second stage for the testing procedure TS-AD.

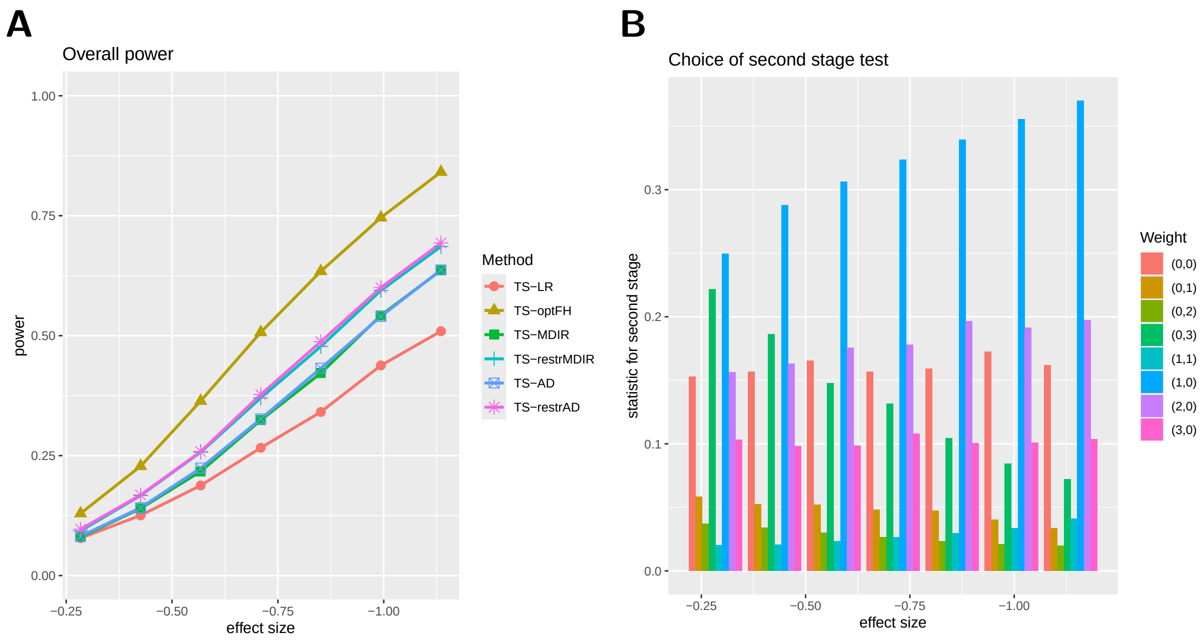

Supplementary Figure S12: A) Power curve for six testing procedures in case of proportional hazards ($(\rho^\star, \gamma^\star) = (3, 0)$). Please note that the two procedures TS-AD and TS-optFH as well as the two procedures OS-MDIR and OS-restrMDIR coincide in this case. B) Relative frequencies of the choice of single weighted tests for the second stage for the testing procedure TS-AD.

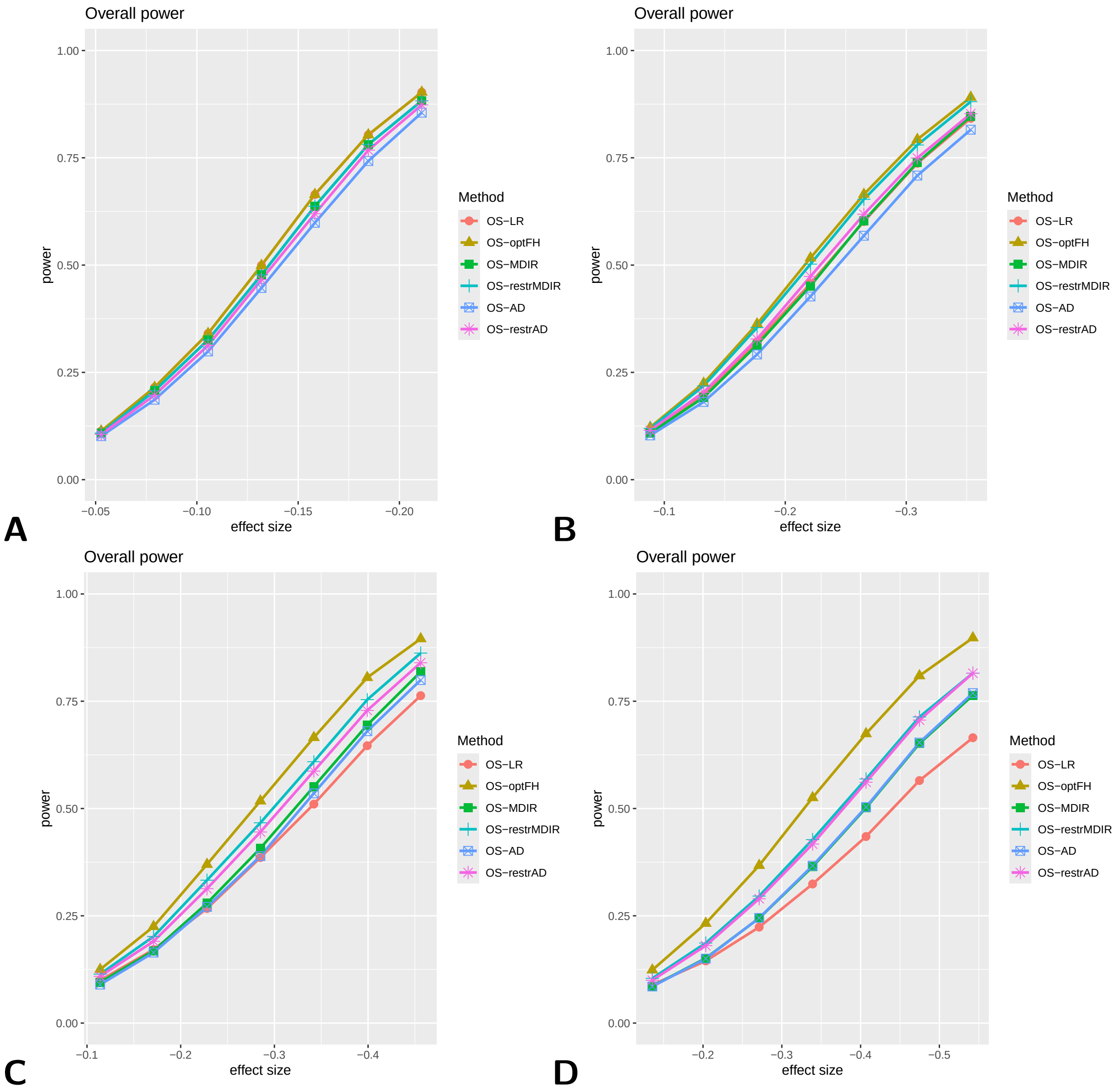

Supplementary Figure S13: A) Power curve for six testing procedures in case of proportional hazards $((\rho^\star, \gamma^\star) = (0, 0))$. B) Power curve for six testing procedures in case of a slight early effect $((\rho^\star, \gamma^\star) = (0, 1))$. C) Power curve for six testing procedures in case of a moderate early effect $((\rho^\star, \gamma^\star) = (0, 2))$. D) Power curve for six testing procedures in case of a pronounced early effect $((\rho^\star, \gamma^\star) = (0, 3))$.

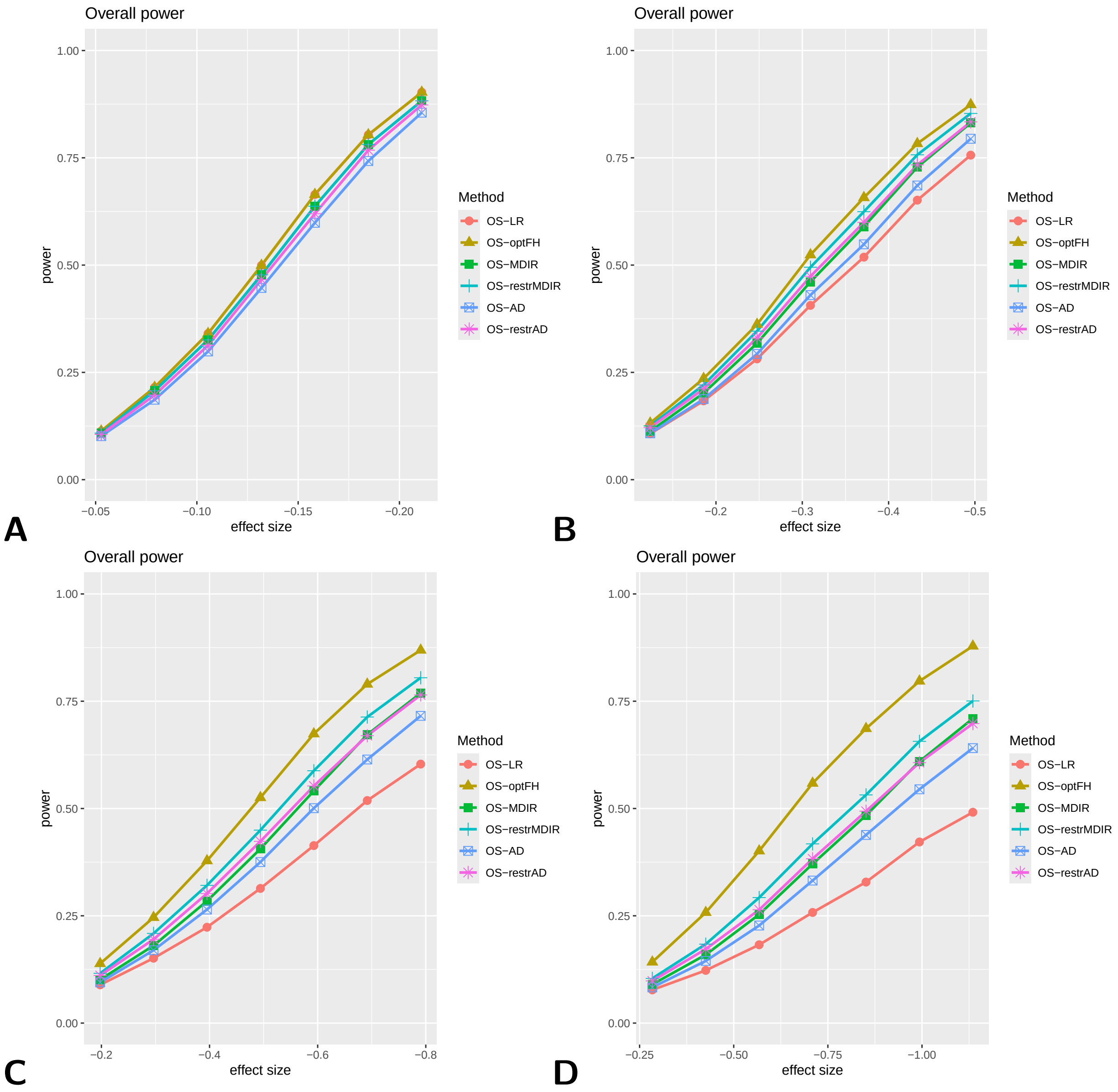

Supplementary Figure S14: A) Power curve for six testing procedures in case of proportional hazards ($(\rho^\star, \gamma^\star) = (0, 0)$). B) Power curve for six testing procedures in case of a slight late effect ($(\rho^\star, \gamma^\star) = (1, 0)$). C) Power curve for six testing procedures in case of a moderate late effect ($(\rho^\star, \gamma^\star) = (2, 0)$). D) Power curve for six testing procedures in case of a pronounced late effect ($(\rho^\star, \gamma^\star) = (3, 0)$).

C.2.2 Model choice based on AIC

Here we show which of the 9 Royston-Parmar spline models is chosen based on their AIC value. As already mentioned, we can see that the AIC selection mechanism prefers models with 0 interior knots for our simulation scenarios. In particular, the model on the hazard scale is preferred. This spline model induces Weibull distributions for both groups.

Effect size	$p = 0$			$p = 1$			$p = 2$		
	hazard	odds	normal	hazard	odds	normal	hazard	odds	normal
$0.4\theta_0$	0.7892	0.0224	0.0001	0.0799	0.0243	0.0227	0.0233	0.0141	0.0241
$0.6\theta_0$	0.7807	0.0252	0.0001	0.0816	0.0273	0.0254	0.022	0.0143	0.0235
$0.8\theta_0$	0.7728	0.0276	0.0001	0.0806	0.0243	0.0263	0.0255	0.0166	0.0262
θ_0	0.7774	0.0284	0.0001	0.0788	0.0288	0.0253	0.0236	0.0141	0.0234
$1.2\theta_0$	0.777	0.0308	0	0.0755	0.0291	0.0203	0.0247	0.015	0.0276
$1.4\theta_0$	0.7689	0.038	0.0001	0.0754	0.0299	0.0223	0.0235	0.0153	0.0267
$1.6\theta_0$	0.7745	0.0368	0.0001	0.0747	0.0264	0.0244	0.0223	0.0163	0.0245

Supplementary Table S11: For each of the seven effect sizes, this table displays which of the nine Royston-Parmar spline models is rated as the best based on the AIC. As above, p refers to the number of inner knots in the spline model. The empirical rates refer to the total quantity of runs without early stopping of the corresponding simulated trial. This table refers to the scenario $(\rho^\star, \gamma^\star) = (0, 0)$

Effect size	$p = 0$			$p = 1$			$p = 2$		
	hazard	odds	normal	hazard	odds	normal	hazard	odds	normal
$0.4\theta_0$	0.763	0.0314	0.0001	0.0827	0.0262	0.0321	0.0219	0.0169	0.0257
$0.6\theta_0$	0.7614	0.0362	0	0.0799	0.0291	0.0347	0.0231	0.0131	0.0226
$0.8\theta_0$	0.7483	0.0412	0	0.0836	0.0291	0.0338	0.0239	0.0148	0.0253
$1\theta_0$	0.7332	0.0479	0.0001	0.0874	0.032	0.0365	0.0238	0.0149	0.0242
$1.2\theta_0$	0.7192	0.054	0.0005	0.0906	0.0338	0.0373	0.0221	0.0156	0.027
$1.4\theta_0$	0.7104	0.058	0.0003	0.0918	0.0362	0.0409	0.0247	0.0128	0.0248
$1.6\theta_0$	0.6998	0.063	0.0003	0.0936	0.0321	0.0416	0.0249	0.0162	0.0284

Supplementary Table S12: For each of the seven effect sizes, this table displays which of the nine Royston-Parmar spline models is rated as the best based on the AIC. As above, p refers to the number of inner knots in the spline model. The empirical rates refer to the total quantity of runs without early stopping of the corresponding simulated trial. This table refers to the scenario $(\rho^\star, \gamma^\star) = (1, 0)$

Effect size	$p = 0$			$p = 1$			$p = 2$		
	hazard	odds	normal	hazard	odds	normal	hazard	odds	normal
$0.4\theta_0$	0.7595	0.0335	0.0001	0.0828	0.0326	0.035	0.0193	0.014	0.0232
$0.6\theta_0$	0.7429	0.042	0	0.0836	0.0357	0.0368	0.0214	0.0134	0.0242
$0.8\theta_0$	0.7213	0.0518	0.0003	0.0812	0.0395	0.0429	0.0219	0.0157	0.0255
$1\theta_0$	0.6932	0.064	0.0001	0.094	0.0391	0.0489	0.0227	0.0154	0.0226
$1.2\theta_0$	0.6595	0.0711	0	0.0985	0.047	0.0517	0.0267	0.0167	0.0288
$1.4\theta_0$	0.6348	0.0833	0.0002	0.105	0.048	0.0517	0.0268	0.0191	0.031
$1.6\theta_0$	0.6007	0.0943	0.0001	0.1136	0.0533	0.0598	0.0297	0.0174	0.031

Supplementary Table S13: For each of the seven effect sizes, this table displays which of the nine Royston-Parmar spline models is rated as the best based on the AIC. As above, p refers to the number of inner knots in the spline model. The empirical rates refer to the total quantity of runs without early stopping of the corresponding simulated trial. This table refers to the scenario $(\rho^\star, \gamma^\star) = (2, 0)$

Effect size	$p = 0$			$p = 1$			$p = 2$		
	hazard	odds	normal	hazard	odds	normal	hazard	odds	normal
$0.4\theta_0$	0.7586	0.0323	0	0.0765	0.0351	0.0359	0.0223	0.0147	0.0246
$0.6\theta_0$	0.7262	0.0461	0.0001	0.0827	0.0369	0.0435	0.0236	0.0167	0.0243
$0.8\theta_0$	0.7094	0.053	0.0001	0.0851	0.0414	0.0426	0.0234	0.0183	0.0266
$1\theta_0$	0.6718	0.0697	0.0001	0.0954	0.0451	0.0496	0.0233	0.0175	0.0274
$1.2\theta_0$	0.6471	0.074	0.0003	0.0958	0.0522	0.058	0.0267	0.02	0.0259
$1.4\theta_0$	0.6214	0.0853	0.0004	0.1007	0.0557	0.0589	0.0288	0.0216	0.0273
$1.6\theta_0$	0.569	0.1016	0.0002	0.1132	0.0631	0.0644	0.0331	0.0229	0.0324

Supplementary Table S14: For each of the seven effect sizes, this table displays which of the nine Royston-Parmar spline models is rated as the best based on the AIC. As above, p refers to the number of inner knots in the spline model. The empirical rates refer to the total quantity of runs without early stopping of the corresponding simulated trial. This table refers to the scenario $(\rho^\star, \gamma^\star) = (3, 0)$

Effect size	$p = 0$			$p = 1$			$p = 2$		
	hazard	odds	normal	hazard	odds	normal	hazard	odds	normal
$0.4\theta_0$	0.7827	0.0209	0	0.0804	0.0256	0.0226	0.0246	0.0155	0.0276
$0.6\theta_0$	0.7762	0.0189	0	0.0864	0.0259	0.0215	0.0274	0.0163	0.0274
$0.8\theta_0$	0.7787	0.0189	0	0.0861	0.0244	0.0224	0.0259	0.0163	0.0273
$1\theta_0$	0.7788	0.0212	0	0.0903	0.0249	0.0176	0.0252	0.0142	0.0279
$1.2\theta_0$	0.7861	0.0182	0	0.0875	0.0229	0.0183	0.0236	0.0176	0.0258
$1.4\theta_0$	0.7889	0.0158	0	0.0836	0.0249	0.0135	0.0298	0.0132	0.0303
$1.6\theta_0$	0.7798	0.0179	0	0.0913	0.0253	0.0137	0.029	0.0134	0.0297

Supplementary Table S15: For each of the seven effect sizes, this table displays which of the nine Royston-Parmar spline models is rated as the best based on the AIC. As above, p refers to the number of inner knots in the spline model. The empirical rates refer to the total quantity of runs without early stopping of the corresponding simulated trial. This table refers to the scenario $(\rho^\star, \gamma^\star) = (0, 1)$

Effect size	$p = 0$			$p = 1$			$p = 2$		
	hazard	odds	normal	hazard	odds	normal	hazard	odds	normal
$0.4\theta_0$	0.7812	0.0194	0	0.0863	0.0284	0.0201	0.0254	0.0143	0.0248
$0.6\theta_0$	0.7752	0.0172	0	0.0887	0.0294	0.0223	0.0238	0.0159	0.0275
$0.8\theta_0$	0.7659	0.0177	0	0.0969	0.0306	0.0208	0.0238	0.0165	0.0279
$1\theta_0$	0.7668	0.0148	0	0.1012	0.0294	0.0204	0.024	0.0165	0.0269
$1.2\theta_0$	0.7636	0.0145	0	0.1015	0.0294	0.0162	0.0278	0.0192	0.0278
$1.4\theta_0$	0.7588	0.014	0	0.1107	0.0309	0.0148	0.0268	0.0173	0.0267
$1.6\theta_0$	0.7526	0.0123	0	0.1194	0.0271	0.015	0.0306	0.0174	0.0256

Supplementary Table S16: For each of the seven effect sizes, this table displays which of the nine Royston-Parmar spline models is rated as the best based on the AIC. As above, p refers to the number of inner knots in the spline model. The empirical rates refer to the total quantity of runs without early stopping of the corresponding simulated trial. This table refers to the scenario $(\rho^\star, \gamma^\star) = (0, 2)$

Effect size	$p = 0$			$p = 1$			$p = 2$		
	hazard	odds	normal	hazard	odds	normal	hazard	odds	normal
$0.4\theta_0$	0.7807	0.0142	0	0.0861	0.0289	0.0226	0.0233	0.0168	0.0274
$0.6\theta_0$	0.7706	0.0185	0	0.088	0.0307	0.0254	0.0244	0.0164	0.026
$0.8\theta_0$	0.7672	0.0178	0	0.0964	0.0309	0.0233	0.0229	0.0186	0.0229
$1\theta_0$	0.7573	0.0183	0.0001	0.0984	0.0351	0.0228	0.0259	0.0174	0.0247
$1.2\theta_0$	0.7571	0.0152	0	0.1066	0.0368	0.0181	0.0227	0.0175	0.0261
$1.4\theta_0$	0.7528	0.0159	0	0.1123	0.0383	0.0195	0.022	0.0154	0.0239
$1.6\theta_0$	0.731	0.0151	0	0.1192	0.0413	0.0216	0.0236	0.0228	0.0254

Supplementary Table S17: For each of the seven effect sizes, this table displays which of the nine Royston-Parmar spline models is rated as the best based on the AIC. As above, p refers to the number of inner knots in the spline model. The empirical rates refer to the total quantity of runs without early stopping of the corresponding simulated trial. This table refers to the scenario $(\rho^\star, \gamma^\star) = (0, 3)$

C.2.3 Modelwise choice of test statistics in the second stage

For the three deviation types from the main manuscript, we also show how the choice of the second stage test is distributed for each of the nine spline models. As already mentioned, models with a higher number of inner knots seem to be better suited to choose an appropriate test in late effects settings. Because of that, one should maybe restrict the set of models to a set with a higher number of inner knots if a possible late effect is anticipated.

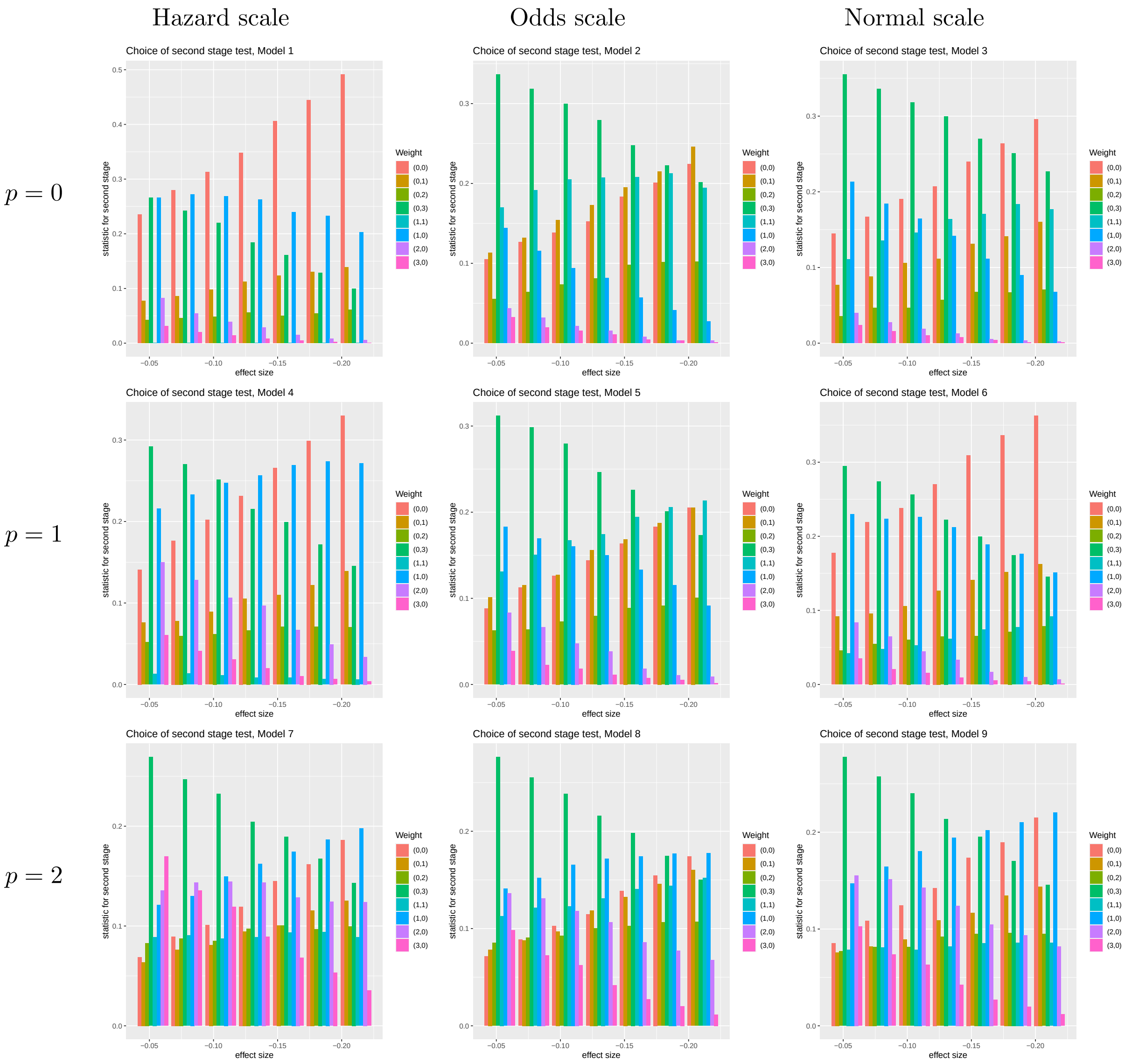

Supplementary Figure S15: Choices of the log-rank test statistic with Fleming-Harrington weights for our nine different Royston-Parmar spline models. The rates refer to the total quantity of simulation runs in which the corresponding simulated trial proceeded to a second stage (i.e. no early termination). The plots are arranged in a grid where the columns refer to different scales and the rows to different numbers of interior knots p. These figures refer to the deviation type $(\rho^\star, \gamma^\star) = (0, 0)$, i.e. proportional hazards.

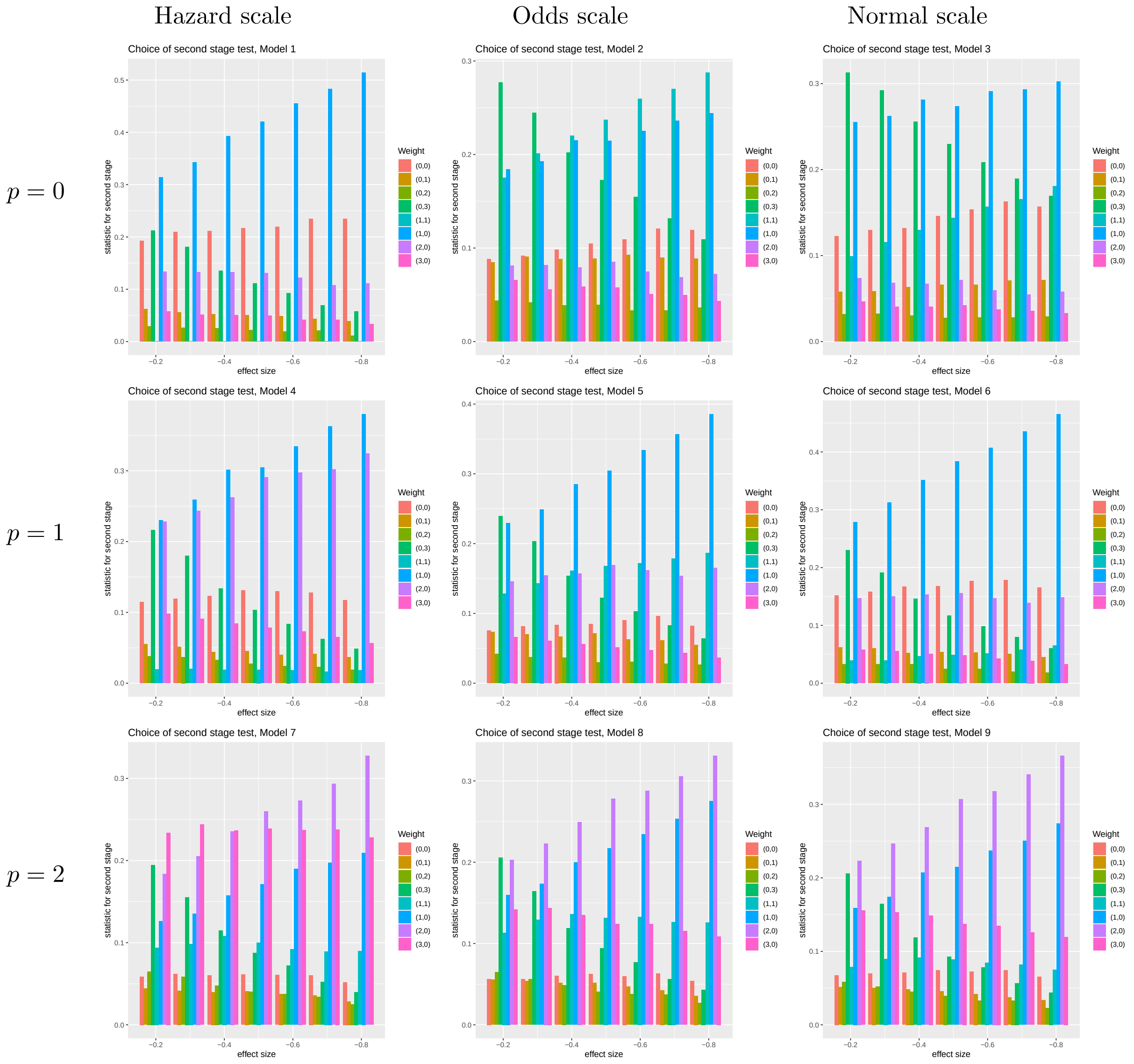

Supplementary Figure S16: Choices of the log-rank test statistic with Fleming-Harrington weights for our nine different Royston-Parmar spline models. The rates refer to the total quantity of simulation runs in which the corresponding simulated trial proceeded to a second stage (i.e. no early termination). The plots are arranged in a grid where the columns refer to different scales and the rows to different numbers of interior knots p. These figures refer to the deviation type $(\rho^\star, \gamma^\star) = (2, 0)$, i.e. a late effects scenario.

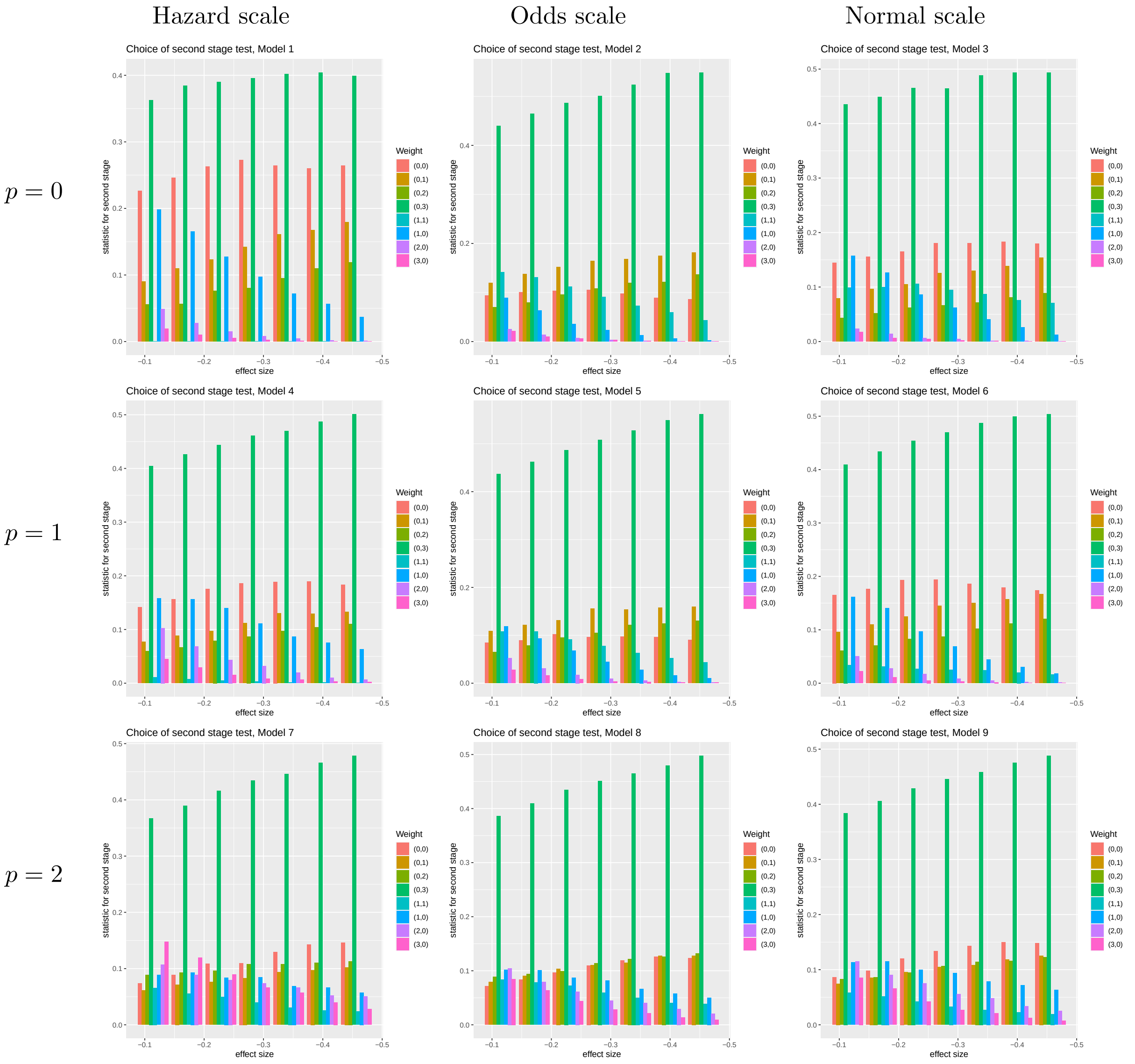

Supplementary Figure S17: Choices of the log-rank test statistic with Fleming-Harrington weights for our nine different Royston-Parmar spline models. The rates refer to the total quantity of simulation runs in which the corresponding simulated trial proceeded to a second stage (i.e. no early termination). The plots are arranged in a grid where the columns refer to different scales and the rows to different numbers of interior knots p. These figures refer to the deviation type $(\rho^\star, \gamma^\star) = (0, 2)$, i.e. an early effects scenario.

References

[1] P. Bauer and F. Koenig. The reassessment of trial perspectives from interim data—a critical view. *Statistics in medicine*, 25(1):23–36, 2006.

[2] M. Brendel, A. Janssen, C.-D. Mayer, and M. Pauly. Weighted logrank permutation tests for randomly right censored life science data. *Scandinavian Journal of Statistics*, 41(3):742–761, 2014.

[3] M. F. Danzer, A. Faldum, T. Simon, B. Hero, and R. Schmidt. Confirmatory adaptive group sequential designs for clinical trials with multiple time-to-event outcomes in markov models, 2023.

[4] M. Ditzhaus and M. Pauly. Wild bootstrap logrank tests with broader power functions for testing superiority. *Computational statistics & data analysis*, 136:1–11, 2019.

[5] C. Jackson. flexsurv: A platform for parametric survival modeling in R. *Journal of Statistical Software*, 70(8):1–33, 2016.

[6] R. H. Jones, A. Casbard, M. Carucci, C. Cox, R. Butler, F. Alchami, T.-A. Madden, C. Bale, P. Bezecny, J. Joffe, et al. Fulvestrant plus capivasertib versus placebo after relapse or progression on an aromatase inhibitor in metastatic, oestrogen receptor-positive breast cancer (faktion): a multicentre, randomised, controlled, phase 2 trial. *The Lancet Oncology*, 21(3):345–357, 2020.

[7] D. Magirr and C.-F. Burman. Modestly weighted logrank tests. *Statistics in medicine*, 38(20):3782–3790, 2019.

[8] D. Magirr and C.-F. Burman. The MaxCombo Test Severely Violates the Type I Error Rate. *JAMA Oncology*, 9(4):571–572, 04 2023.

[9] P. Royston and M. K. Parmar. Flexible parametric proportional-hazards and proportional-odds models for censored survival data, with application to prognostic modelling and estimation of treatment effects. *Statistics in medicine*, 21(15):2175–2197, 2002.

[10] G. Wassmer. Planning and analyzing adaptive group sequential survival trials. *Biometrical Journal*, 48(4):714–729, 2006.
